# Bayesian spatio-temporal analysis of malaria prevalence in children between 2 and 10 years of age in Gabon

**DOI:** 10.1186/s12936-024-04880-8

**Published:** 2024-02-23

**Authors:** Fabrice Mougeni, Bertrand Lell, Ngianga-Bakwin Kandala, Tobias Chirwa

**Affiliations:** 1 Division of Epidemiology and Biostatistics, School of Public Health, University of the Witwatersrand, Johannesburg, 2193 South Africa; 2 Centre de Recherches Médicales de Lambaréné, P.O. Box 242, Lambaréné, Gabon; 3 Department of Medicine I, Division of Infectious Diseases and Tropical Medicine, Medical University of Vienna, 1090 Vienna, Austria; 4 Department of Epidemiology and Biostatistics, Western Centre for Public Health and Family Medicine, Schulich School of Medicine & Dentistry, Western University, London, Canada

**Keywords:** Small area, Bayesian analysis, Environmental factors, INLA, SPDE

## Abstract

**Background:**

Gabon still bears significant malaria burden despite numerous efforts. To reduce this burden, policy-makers need strategies to design effective interventions. Besides, malaria distribution is well known to be related to the meteorological conditions. In Gabon, there is limited knowledge of the spatio-temporal effect or the environmental factors on this distribution. This study aimed to investigate on the spatio-temporal effects and environmental factors on the distribution of malaria prevalence among children 2–10 years of age in Gabon.

**Methods:**

The study used cross-sectional data from the Demographic Health Survey (DHS) carried out in 2000, 2005, 2010, and 2015. The malaria prevalence was obtained by considering the weighting scheme and using the space–time smoothing model. Spatial autocorrelation was inferred using the Moran’s I index, and hotspots were identified with the local statistic Getis-Ord General Gi. For the effect of covariates on the prevalence, several spatial methods implemented in the Integrated Nested Laplace Approximation (INLA) approach using Stochastic Partial Differential Equations (SPDE) were compared.

**Results:**

The study considered 336 clusters, with 153 (46%) in rural and 183 (54%) in urban areas. The prevalence was highest in the Estuaire province in 2000, reaching 46%. It decreased until 2010, exhibiting strong spatial correlation (P < 0.001), decreasing slowly with distance. Hotspots were identified in north-western and western Gabon. Using the Spatial Durbin Error Model (SDEM), the relationship between the prevalence and insecticide-treated bed nets (ITNs) coverage was decreasing after 20% of coverage. The prevalence in a cluster decreased significantly with the increase per percentage of ITNs coverage in the nearby clusters, and per degree Celsius of day land surface temperature in the same cluster. It slightly increased with the number of wet days and mean temperature per month in neighbouring clusters.

**Conclusions:**

In summary, this study showed evidence of strong spatial effect influencing malaria prevalence in household clusters. Increasing ITN coverage by 20% and prioritizing hotspots are essential policy recommendations. The effects of environmental factors should be considered, and collaboration with the national meteorological department (DGM) for early warning systems is needed.

**Supplementary Information:**

The online version contains supplementary material available at 10.1186/s12936-024-04880-8.

## Background

In recent years, epidemiological data have highlighted the global impact of malaria. This has led to an estimated of 241 million cases and 627,000 deaths attributed to the disease worldwide [1]. In Africa, the sub-Saharan region accounts for approximately 90% of the malaria cases and mortality with *Plasmodium falciparum*. Gabon is one of the African countries with a high prevalence of malaria among children under five years of age [2], with high impact on population health and socio-economic levels. Following the sustainable development goals for good health and well-being of Gabon, there is need to reduce the mortality rate of children under-five from 41 to 2.6% [3]. To contribute towards such a goal, reducing the burden of malaria is an important point to target since, in Gabon, malaria is the main cause of consulting, hospitalization, and deaths in children [4]. To contribute towards this objective, Gabon, through its national programme, is committed in the initiative of taking action against malaria to mitigate the substantial impact of malaria in the coming years. In the previous years, several control strategies were already implemented. This included (i) vector control, such as the distribution of insecticide-treated nets (ITNs), long-lasting insecticidal nets (LLINs), and indoor residual spraying (IRS); (ii) chemoprevention; (iii) diagnosis and treatment, such as the introduction of rapid diagnostic tests (RDTs) and microscopy help diagnose malaria quickly and accurately, and the use of anti-malarial drugs according to national treatment guidelines help cure infected individuals and prevent the spread of drug-resistant malaria strains; (iv) community engagement and education, such as health promotion by educating communities about malaria transmission, prevention measures, and the importance of seeking prompt treatment. Community participation where local communities are involved in malaria control programmes, fostering ownership and sustainability; (v) research and surveillance; or (vi) the integrated approach: implementing multiple interventions simultaneously (e.g., using ITNs alongside IRS) to create synergistic effects and improve overall effectiveness. For many of them, their effectiveness was reported in different areas of Gabon [5, 6].

From previous studies, children under 5 years of age were shown to be the most affected [7]. Recently, some studies suggested a shift in the high-risk age group [2]. However, the *P. falciparum* parasite rate (PfPR) in children between 2 and 10 years old is used as the standardized measure for malaria endemicity. In fact, three reasons have driven this choice when considering malaria intensity transmission [8]: (i) the constant PfPR in this age group; (ii) the PfPR may be least influenced by drug treatment and not affected by immunity and it’s reflecting stationary state predicted from mathematical model in terms of clinical malaria in this group; (iii) the PfPR is aligned with the historical approaches of endemicity statement. PfPR is generally considered as the best indicator of the intensity of the transmission, given its association with incidence of clinical malaria episodes [9].

In Gabon, some studies observed a decline in the prevalence of malaria, which was noticed after the improvement of the anti-malarial policy since 2002, through interventions including impregnated bed nets, and artemisinin-based combination therapy (ACT) [6]. A cross-sectional survey conducted in Libreville, Port-Gentil, Melen and Oyem, based on health facilities, showed that between 2005 and 2008, the prevalence fell from 31 to 18% for children under 11 years of age. Another study conducted in Libreville in 2009, using a large number of febrile children, showed a decrease of malaria between 2000 and 2008 [2, 10]. After 2008, several studies conducted separately suggested there was no evidence of decline of the prevalence up to 2012 or 2013 [5, 11, 12]. However, the conclusion has remained in some way ambiguous partly because of the scarcity of the available data. Furthermore, the factors often considered as related to the prevalence in Gabon were the level of education, the type of house, the open water body, and the source of drinking water. These were mainly investigated at a local scale [5, 10, 12]. However, some factors act differently on the vector, the parasite and the host-vector interaction, such as the temperature. The optimal temperature is between 25 and 27 °C, and is important for the duration of the development of the parasite in the vector [13]. Below 16 °C, parasites stop growing, and above 28 °C or 30 °C there is a fast decrease of *Anopheles* prevalence [14, 15]. The mean, minimal and maximal day and night land surface temperature are widely used in such analyses. Moreover, rainfall, humidity and arid conditions, are also implied in the egg laying, multiplication and the survival of the vector. The land coverage can also be important since it can locate mosquito abundance and allow the interaction between vector and host [13].

In the context of malaria transmission and other infectious diseases, the assumption of independence among observations may not be met. Factors such as environmental conditions, vector behaviours, and human interactions can create spatial dependencies, where neighbouring households or clusters may influence each other's health outcomes due to shared exposures or similar environmental conditions. Considering spatial dependency with time is essential to gain a more accurate understanding of the distribution and dynamics of malaria prevalence. By incorporating spatio-temporal effects, it is possible to account for the influence of nearby locations and the temporal evolution of malaria distribution. Bayesian methods are well-suited for this type of analysis due to their flexibility. For example, they were used in a study in Rwanda for the estimation of incidence without including any covariates [16], and in Burundi, Uganda, Nigeria and Tanzania, to describe the relationship between malaria distribution, climatic factors, and anti-vector interventions [14, 17].

Despite progress in malaria vaccine developments, there is need to better understand malaria and its risk factors using different approaches. These include incorporating spatial components and environmental factors in the modelling in order to understand disease prevalence patterns and to help in the formulation of research hypothesis for future research and to identify appropriate interventions. This study aimed to provide a description of the spatio-temporal distribution of malaria prevalence (PfPR) over time and its relationship with environmental factors adjusted for known anti-vector intervention (ITNs coverage), population density and household wealth index for children between 2–10 years based on data from household clusters.

## Methods

### Study design

This study used secondary data from the DHS program, consisting of a series of cross-sectional surveys carried out on 4 time points (2000, 2005, 2010 and 2015) to provide a representative estimate at the national or regional level [19]. The malaria measure in these data was the prevalence of *P. falciparum* malaria (PfPR) in children between 2–10 years of age per cluster as measured by rapid diagnostic tests or microscopy test. Although the DHS program continues to collect data after 2015, only these time points were used, since no recent relevant data were available for Gabon after 2015 due to the emergence of COVID-19 infection during the year 2020.

### Study site

**Fig. 1:**
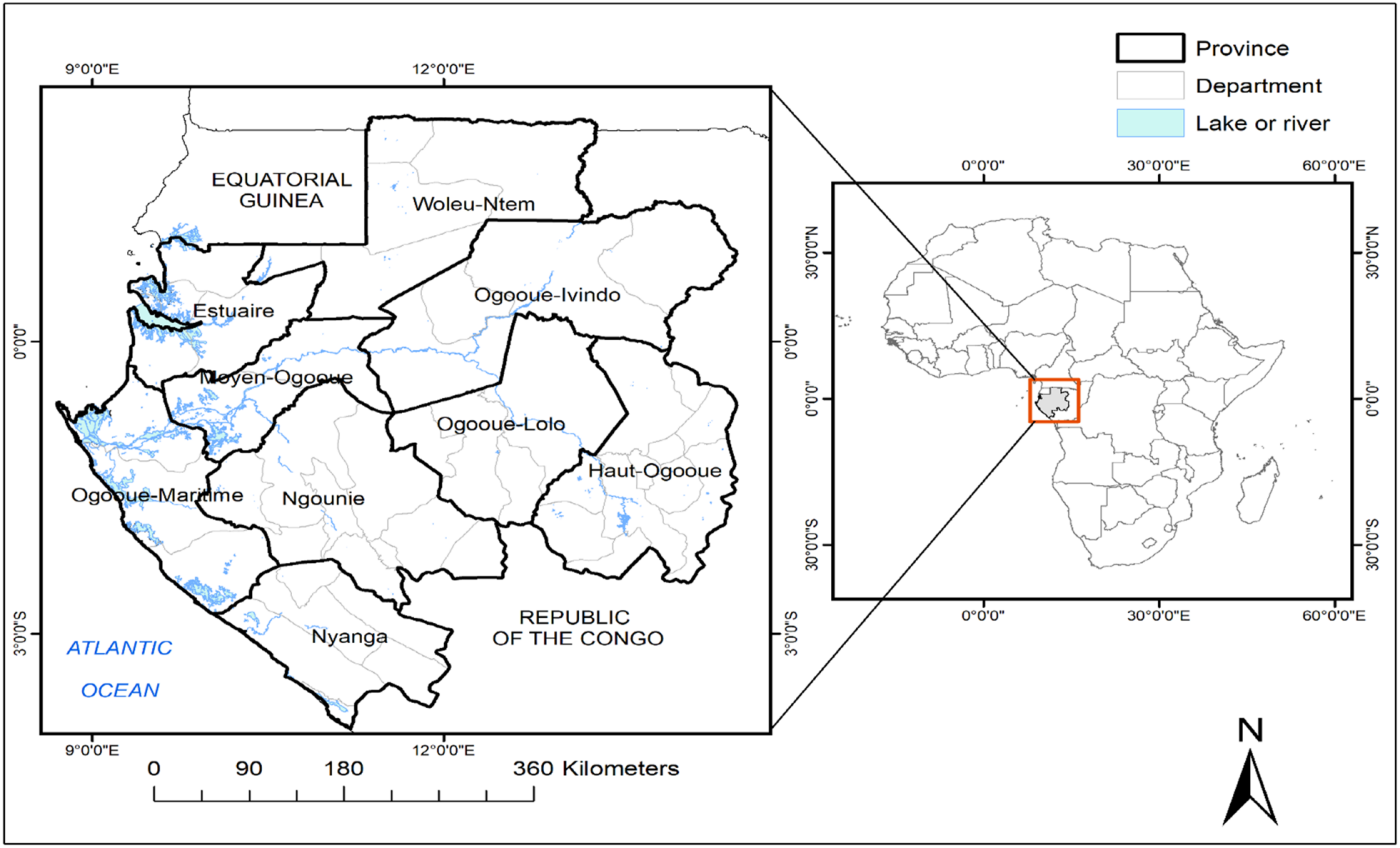
The Map of Gabon

The DHS study was conducted in Gabon which is situated on the West coast of Central Africa. It is bordered by the Atlantic Ocean at the west part, Equatorial Guinea and Cameroon in the north, and Republic of the Congo in south crossed by the Equator. The rainy season extends from October to May with a dry season from June to August [20]. Gabon has a total of 9 provinces with Libreville as its capital city. The whole area is subdivided in 49 departments with an estimated total population of 2.1 million (2019) (Fig. 1). The study was conducted in both urban and rural areas.

### Data sources and sampling

Information on the sampling frame was obtained from the 2000 report of a health survey of the Gabonese direction nationale des statistiques (DGS) [21], namely the number of households in each cluster and number of clusters in each residence and resident area. The data were made available upon request by DHS program. The stratified multi-stage sampling method was used for conducting the survey [22]. The country was divided into regions consisting of provinces and two major towns (Libreville and Port-Gentil). Within each region the primary sampling units were clusters of households stratified by area (urban or rural), totaling 20 strata. A random number of households was selected within each chosen cluster, their sampling weight and their centroid calculated. The number of households was in the range of 100–300 for each cluster. For all the surveys, 336 clusters were sampled with 5 to 30 households per cluster. Spatial data were available for each cluster centroid. For this study, Libreville and Port-Gentil were excluded due to the missing the data on malaria prevalence. All the covariates used here were coming from the raster data and were re-projected to the same standard-based World Geodetic System 1984, then resampled to a 5 × 5 km spatial resolution. These rasters and the GPS shapefile were imported into R to extract raster value. More details can be found in Mayala et al*.* [22].

### Statistical analysis

#### Descriptive analysis

Since for each cluster of households, the PfPR was provided by year, the mean and standard deviation were used to get a summary of the distribution of the prevalence in each province by year. The PfPR for each cluster time was plotted in order to observe the prevalence within and between clusters.

#### Bayesian inference and computation with INLA

The Bayesian model presents several advantage over the frequentist approach. Firstly, in the context of this study the data contain hierarchichal structure and potential dependency, secondly the study is dealing with secondary data and the small areas context. In that situation there is need to quantify uncertainty and to remove variability through spatial smoothing in order to obtain a good estimation of the prevalence. While frequentist can also deal with spatial analysis presenting the advantage of simple interpretation in terms of decision through the use of p-value, Bayesian modelling offers advantages in its flexibility, incorporation of prior information, robustness in small samples, uncertainty quantification, and ability to handle complex spatial structures. The computation was made using the Integrated Nested Laplace Approximation (INLA) approach instead of the usual MCMC method. The Bayesian inference helps to provide a distribution of the parameters ($\theta$) given the data ($y$) called posterior distribution ($p\left(\theta |y\right)$), corresponding approximatively to the product of the distribution assigned to this parameter before seeing the data called prior ($p\left(\theta \right)$), and the likelihood (product of the individual probability of ${y}_{i}|\theta$)) using Bayes theorem.

It can be briefly expressed as:

$$p\left(\theta |y\right)\propto p\left(y|\theta \right)\times p\left(\theta \right)$$

The marginal posterior distribution was obtained using an approach suggested by Rue et al. [23], called INLA. It is based on the latent Gaussian model, the Gaussian Markov Random Field model (GMRF) and the Laplace approximation, with the aim of reducing the computation time by adding the assumption of the conditional dependence of the parameters. The model can be briefly explained as follows: by denoting $x=\left(\omega ,\alpha ,\beta \right)$ as the latent fields, and $\phi =\left({\sigma }_{e}^{2},k,{\sigma }^{2}\right)$ as the hyper-parameters, in order to find the marginal posterior, $p\left({x}_{k}|y\right)$, an approximation of $p\left(\phi |y\right)$ and $p\left({x}_{k}|\phi ,y\right)$ is calculated, since the marginal posterior can be expressed as:

$$p\left({x}_{k}|y\right)=\int p\left({x}_{i}|\phi ,y\right)p\left(\phi |y\right)d\phi$$

Then the Laplace approximation and the nested integration was used to obtain an approximation $\widetilde{p}\left(\phi |y\right)$ of $p\left(\phi |y\right)$, and $\widetilde{p}\left({x}_{i}|\phi ,y\right)$. Lastly, a numerical integration was used to obtain the approximation $\widetilde{p}\left({x}_{k}|y\right)$ of $p\left({x}_{k}|y\right)$ which takes the following form [24, 25]:

$$\widetilde{p}\left({x}_{k}|y\right)={\sum }_{k}\widetilde{p}\left({x}_{i}|{\phi }_{k},y\right)\widetilde{p}\left({\phi }_{k}|y\right){\Delta }_{k}$$

#### Estimation of the prevalence using structured and unstructured spatial effects 

In order to remove some of the variability in the prevalence and to obtain the smoothed prevalence, two random effects were introduced: (1) a spatial unstructured effect modelled as $i.i.d$ errors with $N\left(0,{\tau }_{e}\right)$, and (2) a spatial structured effect with a Intrinsic Conditional Auto-Regressive (ICAR) model. These account for region and neighbourhood effects and are known as BYM or BYM2 models or convolution models. Additionally, an interaction effect to relax the assumption of lack of specific temporal evolution for each cluster was used. Four types of models of interaction were compared. Type 1, specific area time trends interact with random effect of the region without being influenced by neighbours, Type 2, the temporal trend of a specific area is not influenced by its neighbour, Type 3, the spatial dependence or spatial pattern is not changing according to time, and Type 4, spatial patterns correlated in time or temporal trends spatially correlated specific for each cluster. To obtain an improved estimate, which we called the “true” prevalence, the space and time estimate of the prevalence was obtained firstly through the Horvitz and Thompson formula, and then smoothed using the logit of the obtained prevalence. The weight used in the Horvitz formula at the cluster level was obtained by following the formula suggested by DHS, using the available information in the data, and the DHS final report, which are also used in DGS [21] (see Additional file 1: Table S3). In the estimation of the prevalence, the penalized complexity priors (PC) was used since it is less sensitive to the change of parameters, and it can overcome the difficulty of setting a prior for a hierarchical model as noted in the study of Simpson [26] (Table 3).

#### Estimation in areal and geo-statistical data

The estimation of the prevalence was done using two types of data: areal data and geo-statistical data. This was in order to highlight the use of one of this type of data while estimating the prevalence to borrow strength from other provinces. In the areal data, the unit was a polygon representing a province. Due to the small number of the provinces, the analysis was also conducted in the smallest level, which was cluster of households [27]. Each prevalence value of a cluster was considered as a partial realization of a stochastic process, and the spatial dependency was measured by using only the distance. This made the assumption that nearby points have the approximately the same value. This spatial dependency was measured using the well-known covariance function called Matérn covariance function [28]. To deal with the intensive computation due to the size of the variance–covariance matrix carrying the spatial correlation, the stochastic partial differential equation (SPDE) approach was used. This approach allows to find a differential operator on an SPDE which has the solution described by a covariance function specified above. It provides a sparse precision matrix by moving from the exact solution (a Gaussian field), to the GMRF, the approximated solution, through meshes (48). This was implemented using INLA and the results are very similar to those obtained by the MCMC method [29] (see Table 3 for the SPDE).

#### Geo-additive model (GAM)

In the assessment of the effect of the covariates and the prevalence of malaria, after performing the spatio-temporal analysis with the linear model or the non-spatial linear model (not presented here), the assumption of linearity between them was relaxed by using the GAM, in order to allow the shape of the relationship to be closely related to the observed data, and the results to be compared between the two approaches. In such a GAM, the relation is described for each continuous variable by a function which may be linear or not [30, 31]. Two approaches were developed. The first one, named replicated model, assumed that the coefficient ρ of AR(1) is zero (see Additional file 1: Fig. S16). In the second one, named space–time correlation model, it is assumed that ρ is not 0, and must, therefore, be estimated (see Table 3).

#### Spatial econometrics models

In order to quantify the spillover effect, that is the way the change of a covariate in a region can affect the prevalence in the same region or in another region, the following spatial econometrics models were used: SLM, SEM, SDM, and the Spatial Durbin Error Model (SDEM) [30, 31] (Table 3). While these models are widely used in econometrics, they are not directly implemented in INLA. To be INLA-compatible, it is suggested to condition on the spatial autocorrelation parameter [29, 32].

#### Spatial autocorrelation and hotspots 

The Moran’s index I was used for detecting the spatial autocorrelation on the outcome, malaria prevalence, at the cluster level. A positive Moran’s statistics close to 1 or a Geary c statistic close to 0 indicated a strong autocorrelation of the prevalence. For identifying hotspots and cold spots, the local indicator of spatial autocorrelation (LISA) was used.

#### Spatial regression with INLA

Variable selection was based on a Gaussian random effect model, the multicollinearity using the (variance inflator factor, VIF) with the cut-off set at 10, and the knowledge of the variables commonly used in the literature. Covariates included in the model study were first available from the data as known variable influencing the distribution of the prevalence, however to be considered in the analysis they were selected in order to avoid redundance effect or variables which are not improving the fit of the model. The contribution of each variable according to the literature was the main criteria of selection. As the objective was also to compare in the model how environmental variables are affecting the distribution in the presence of other variables such as population density, socio-economic factors such as wealth index, and the vector control such as ITNs coverage, these variables were included in the final model and were considered in the selection model presented here (for details see Additional file 1: Table S7—S11). The linear regression was mainly used to see whether the variation observed can be sufficiently explained by the covariates. Hence, the spatial autocorrelation was measured in the residuals. Spatio-temporal models were run as the appropriate method for spatial effect observed. The covariates were split into four sub-models: M1 (model without covariates), M2 (model with only ITNs coverage, population count and wealth index), M3 (only ecological variables) and M4 (the full model). The variation of the spatial parameters was observed in each model. This approach was suggested by Giorgi et al*.* [33]. Spatial analysis was conducted considering each point as a realization of a Gaussian process, thus defining a stochastic process implemented using INLA and SPDE.

## Results

### Descriptive analysis

**Fig. 2:**
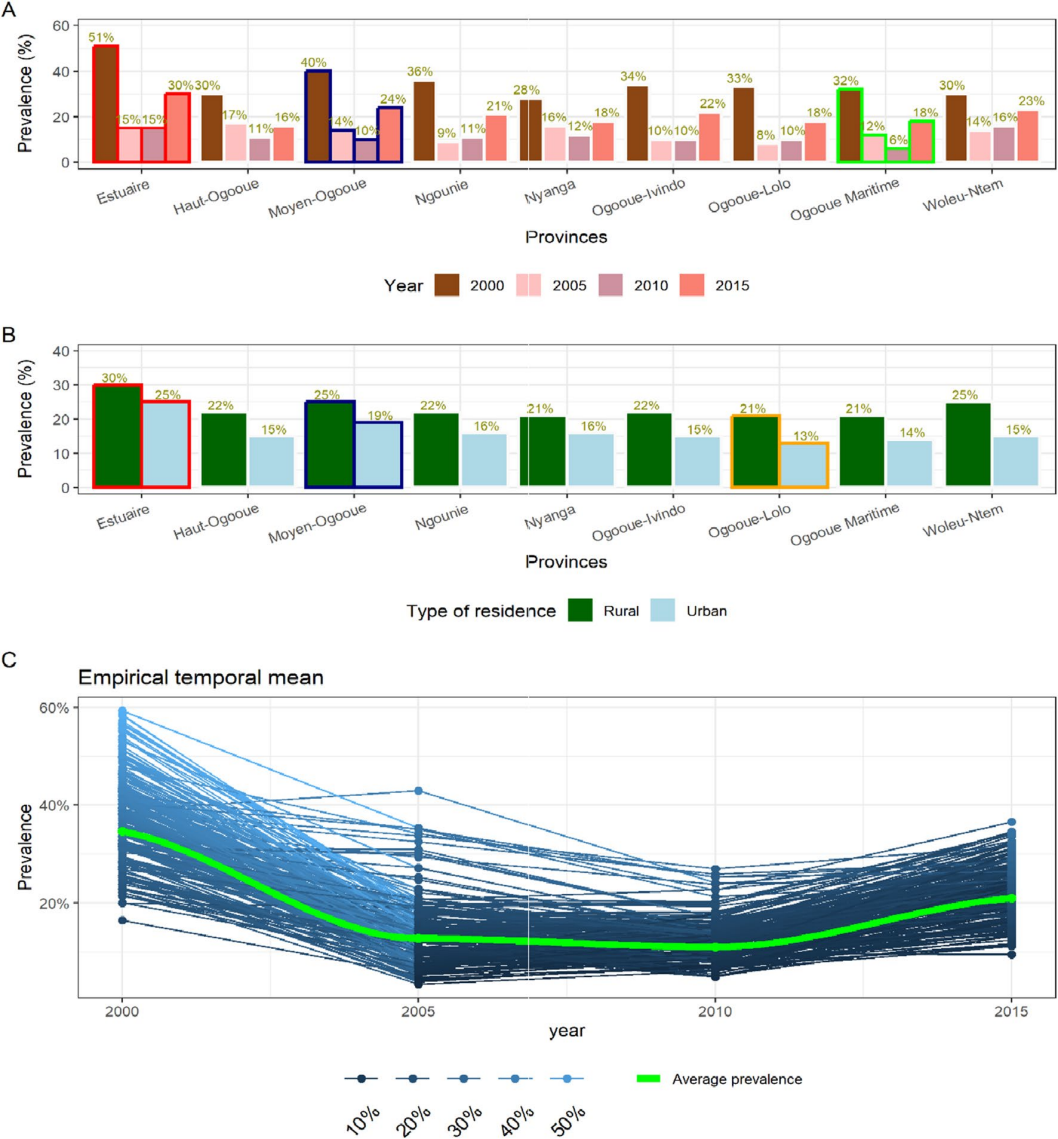
An overview of the distribution of the prevalence. **A** the variation of the prevalence over time by province. The colors red and blue highlight the first two provinces with high prevalence. The green color highlights the province where the prevalence reached the lowest value. **B** distribution of the prevalence by type of residence for each province. **C** prevalence for each cluster over time with the green line showing the overall trend based on polynomial functions

**Fig. 3:**
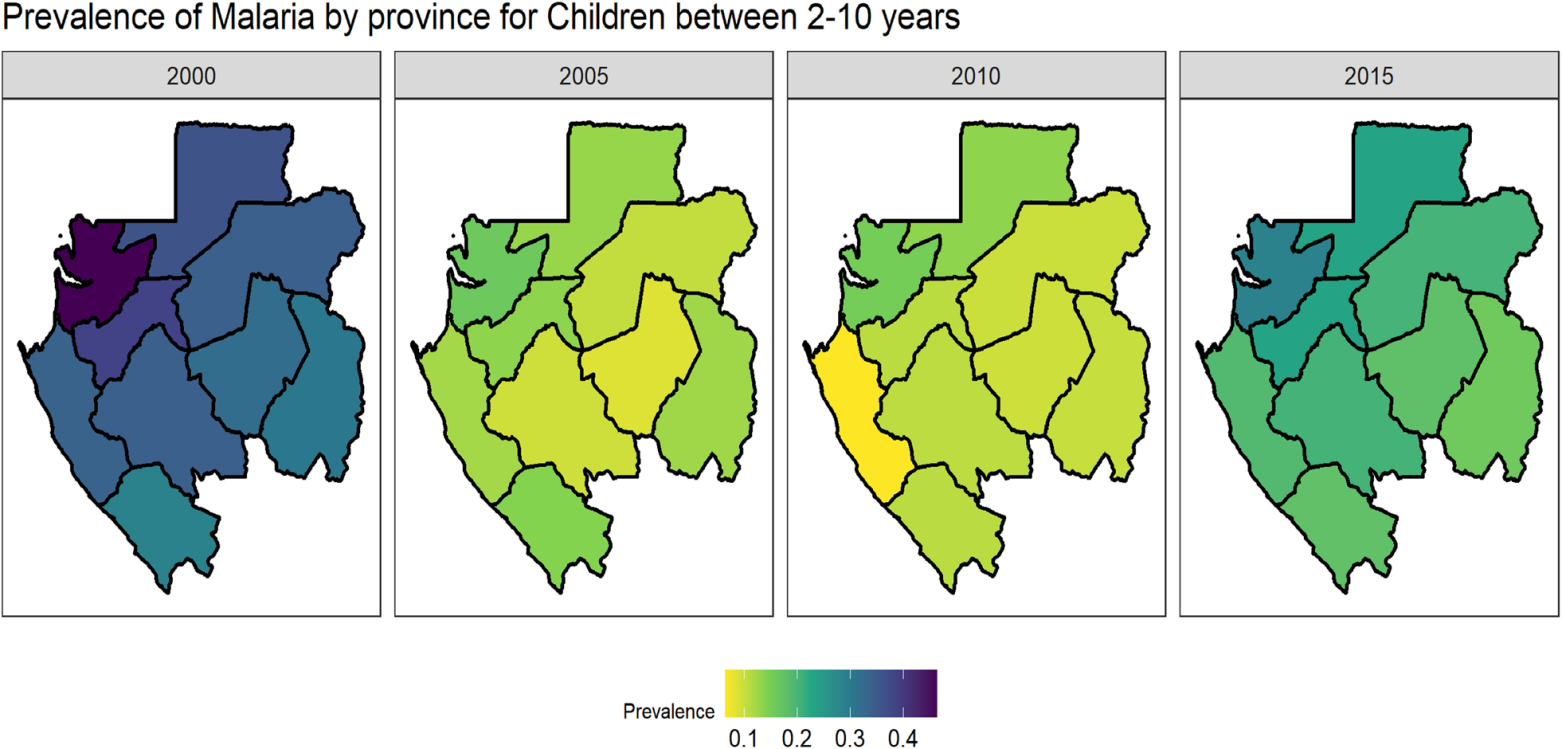
Map of malaria prevalence in Gabon by year (2000, 2005, 2010, 2015). This plot presents the estimate obtained with the smoothing model and survey complexity

This study included 336 clusters, with 153 (46%) clusters in rural areas and 183 (54%) clusters in urban areas. The prevalence of malaria was high in the Estuaire province [29% (± 16%)], followed by Moyen-Ogooue [22% (± 13%)] and Woleu-Ntem [21% (± 10%)]. The lowest prevalence of 17% (± 9%) was found in Haut-Ogooue province. On average, prevalences were similar between the provinces and had large variation. In Fig. 2, panel (A), the prevalence showed a decrease over time from 2000 to 2010 in each province, with a small increase after 2010. In Fig. 3, panel (B) shows that the prevalence was higher in rural areas than in urban ones. Almost all clusters showed this U-shape, as seen in panel C.

It was observed that the mean value of almost all the variables is not changing over time, and only rainfall, population count, ITNs coverage, and aridity are slightly changing. ITNs coverage was the most varying, showing a decrease over time (see Additional file 1: Table S2).

### Space–time estimation of the prevalence

Comparing various models of interactions, the study determined that the type 2 interaction yielded the best results based on DIC. This suggests that the model which shows that the spatial effect does not change over time, and that the temporal effect does not vary across space is the best. The model with interaction exhibited slightly better performance compared to the model without interaction (-145 vs 141 DIC) and demonstrated the highest spatial fraction (41% vs 35%) (see Additional file 1: Table S3 and S4). A comparison between estimates obtained using the smoothing method with and without the sample design revealed a reduction in variance. Hence, the model incorporating the sample design (Eq. (1)) and the smooth method (BYM2 Eq. (2) from Table 1) should be considered to avoid estimates with large variability, as the case was when the sampling design used in the sampling method is not included in the analysis. The analysis identified Estuaire province as having the highest prevalence of malaria throughout the years, followed by Moyen-Ogooue Province (Fig. 3). The prevalence was high in 2000, decreased over time and increased slightly again after 2010, roughly the same in each province. Ogooue-Maritime province was characterized by a very low transmission during the year 2010. This was less than 10%, therefore, characterizing the region as a hypo-endemic area. In general, the whole country remained a meso-endemic area over time with prevalence between 10 and 50%.

**Table 1:** Spatial parameters OLS and GAM with all variables considered as non-linear

Model	Parameter	Spatio-temporal OLS model		GAM model with non-linear variables	
		Mean (SD)	95% CI	Mean (SD)	95% CI
M1	Error	0.000064 (0.0000077)	(0.00005,0.00008)	0.00053 (0.000064)	0.00042;0.00067
	Spatial variance	0.17 (0.036)	(0.11,0.25)	11 (2.2)	7;16
	Range	333 (35)	(270,406)	332 (31)	276;396
	Time coefficient (a)	0.97 (0.0044)	(0.96,0.98)	0.99 (0.0016)	0.98;0.99
M2	Error	0.000026 (0.0000045)	(0.000018,0.000036)	0.00047 (0.000062)	0.00036;0.0006
	Spatial variance	0.055 (0.01)	(0.038,0.077)	8.1 (1.7)	5.2;12
	Range	154 (15)	(128,185)	278 (27)	229;336
	Time coefficient (a)	0.97 (0.0044)	(0.96,0.98)	0.99 (0.0015)	0.99;0.99
M3	Error	0.000071 (0.000012)	(0.00005,0.000095)	0.0006 (0.000082)	0.00045;0.00077
	Spatial variance	0.021 (0.0053)	(0.012,0.033)	10 (2.3)	6.4;15
	Range	153 (21)	(114,197)	342 (36)	279;422
	Time coefficient (a)	0.93 (0.014)	(0.89,0.95)	0.99 (0.0018)	0.98;0.99
M4	Error	0.000023 (0.0000043)	(0.000016,0.000032)	0.00053 (0.000073)	0.0004;0.00068
	Spatial variance	0.044 (0.011)	(0.026,0.068)	8 (1.9)	4.9;12
	Range	142 (17)	(111,176)	290 (33)	230;360
	Time coefficient (a)	0.96 (0.0062)	(0.95,0.97)	0.99 (0.0016)	0.98;0.99

^*^GAM Geo-additive Model; *OLS* Ordinary Least Squared

### Unit-level model

**Fig. 4:**
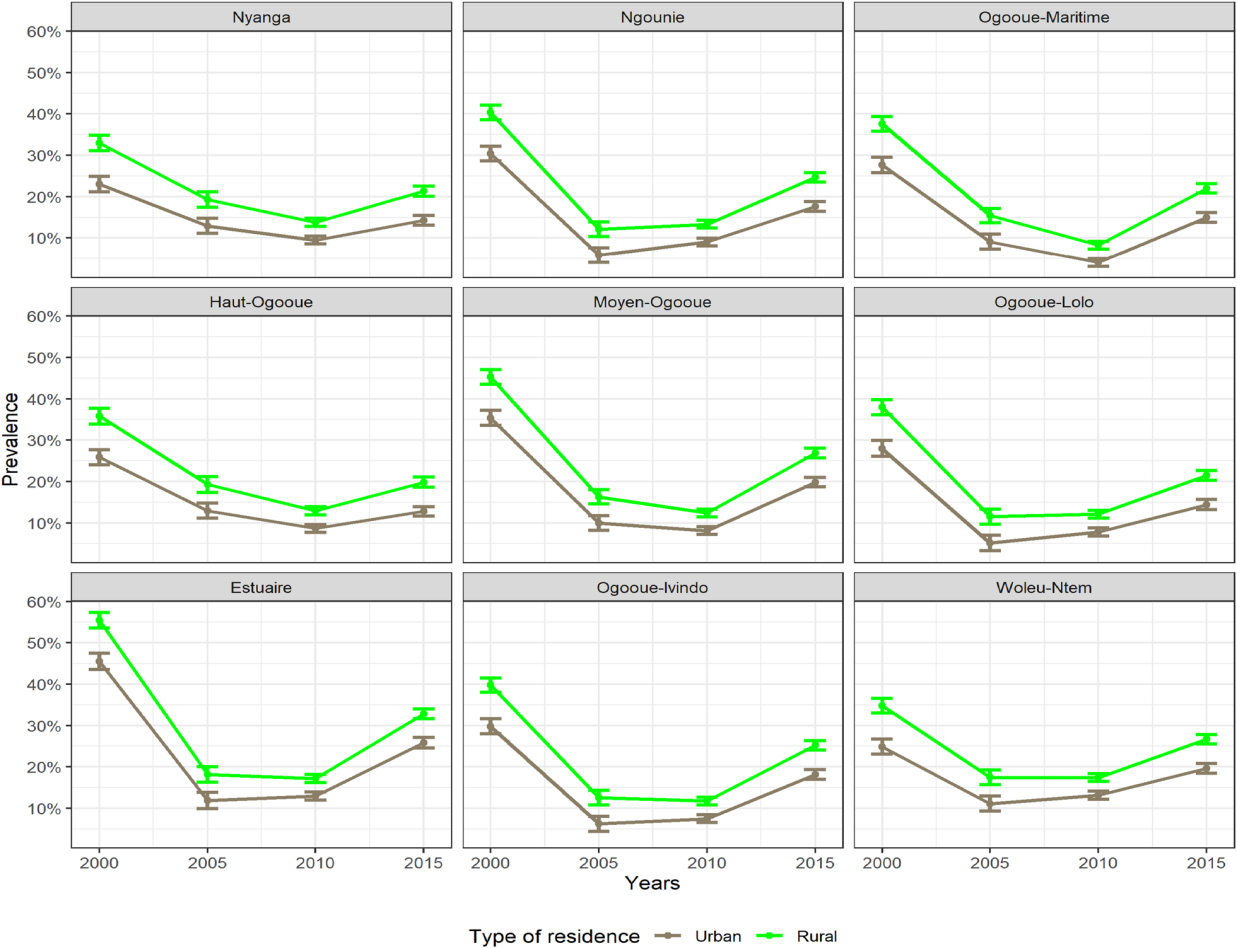
Smoothed prevalence by type of residence obtained from the unit-level model estimated for each year. The bar plotted at each point represent the 95% CI. In contrast to Fig. 2, this is an estimate obtained from a unit-level model

In Fig. 4, the unit-level model using smoothing model shows also that the prevalence of malaria had a U-shape for each province, and each type of residence over time, and high in rural areas by about 7% more than the urban areas. Using this model, the rural areas of the Estuaire province were found to be hyper-endemic areas during the year 2000, while almost all urban areas have low transmission intensity during the year 2010. In general, all the country was meso-endemic until 2015 (see Fig. 4). Compared to the areal model, the length of the credible interval was reduced with the unit-level model. The maximum length of the credible interval went from 12 to 4%, and the minimal length went from 4 to 1% from area-level model to unit-level model, respectively (see Additional file 1: Tables S5 and S6). This was different with the estimation in the Fig. 2 since it was a descriptive analysis.

### Spatial fraction and spatial autocorrelation

The spatial fraction in the prevalence estimated, obtained with the reparametrized BYM model (BYM2) presented in the Table 1 (Eq. (2)), was observed to vary slightly by year. This followed the trend of the estimated malaria prevalence over time (Range: 30%, 46%). There was a statistically significant (p < 0.001) autocorrelation of malaria prevalence in the cluster of the households as the Moran’s index I was positive and close to 1. This was almost constant over time (see Additional file 1: Table S4, Fig. S20).

### Hotspot and coldspot analysis

**Fig. 5:**
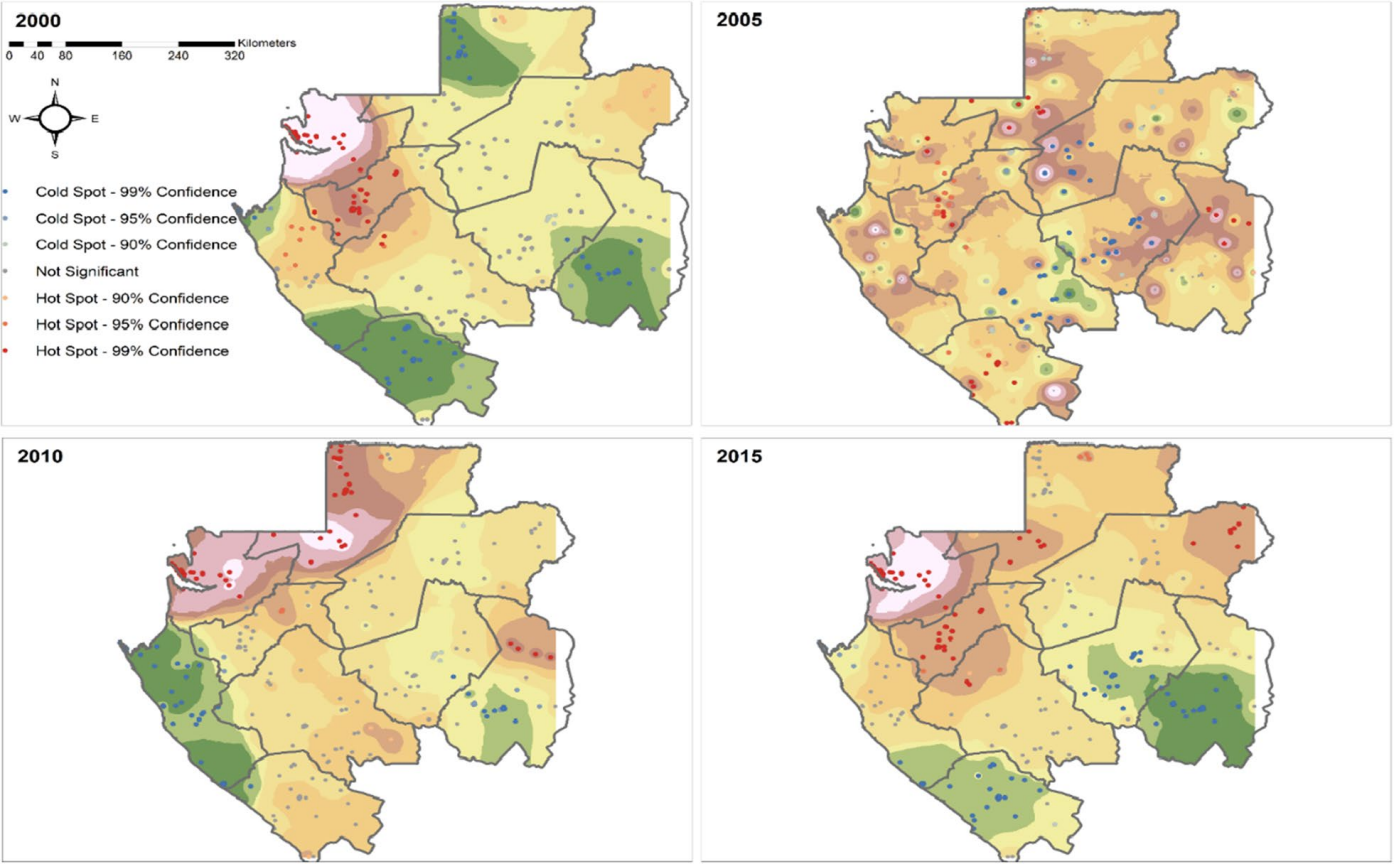
Malaria hotspots and cold spots identification over time in Gabon

As shown in in Fig. 5, using the LISA method, significant hotspots were identified, and the magnitude was varying over time. In the year 2000, a significant cluster of high prevalence was observed in the western part of Gabon at 99% confidence. During 2000 and 2010 the number of hotspots was significantly reduced. However, in the year 2015, almost the same patterns observed in 2000 appeared again. Significant cold spots were found with magnitude changing over time. During the year 2000, the greatest cluster of low prevalence was found in the southern part, followed by the southeast, and the northwest Gabon.

### Model building 

Using the pairwise correlation test, malaria prevalence was negatively associated with many covariates such as ITNs coverage, and night land surface temperature. It was positively associated with mean temperature and wet days. Although minimum and maximum temperature are widely used in the literature, since no evidence of correlation was found, they were removed from the model. However, since there was a strong correlation between minimum temperature and mean temperature, the mean temperature was used in the model to replace these variables in order to have an estimate of the effect of temperature. Based on a mixed random effect model, and the multicollinearity using the VIF (< 10), the following variables were the final variables selected: Proximity to water, all population count, aridity, day land surface temperature, enhanced vegetation index, ITN coverage, mean temperature, wealth index, wet days and rainfall (Additional file 1 for details, Table S8, S9, and S10).

### Multiple linear regression and spatial autocorrelation in the residuals

The linearity of the selected variables and the outcome was assumed looking to the scatter plot and the model was based on the log transformation. Overwhelming evidence of strong spatial autocorrelation in the residuals for each year was found, with only a slight reduction compared to the spatial autocorrelation in the prevalence Additional file 1 for details, Table S12, S13).

### Spatio-temporal models

#### Linear models and GAM

Spatial variation was reduced by 68%, 88%, and 76% for SPTOLS, and by 26%, 9%, and 27% for space–time correlated GAM after adjusting with M2, M3 (environmental variables), and M4 (full model) from M1, respectively (Table 1). The full model, exhibiting the smallest DIC, was considered the most optimal. The value of the range showed a strong spatial correlation decreasing slowly up to 142 km (290 km with GAM). For all the sub-models, the spatial variance was obviously greater than the measure error by more than 95%. The coefficients for time ($\rho$) were almost the same for all the three sub-models. No evidence for the time effect was found since the credible interval contains 0. From AR (1), no effect of time was found since the coefficient for time was relatively high. For the replicated GAM, M3 was better than M2, while for the space–time correlated model, M2 was better than M3 with respect to DIC (see Additional file 1: Table S19).

In the full model, a 1% increase in ITNs coverage and or population density was found to be significantly associated with a 52% reduction (3% reduction) in malaria prevalence. Conversely, a 1-degree increase in night land temperature showed a significant association with a 3% increase in malaria prevalence (see Additional file 1: Table S15). See Eq. (5) and Eq. (5) from Table 3 for spatial linear model and spatial GAM, respectively.

### Spatial econometric models

#### Computing models

Using the Moran index, or the Lagrange multiplier (LM), no evidence of spatial dependence was observed (Additional file 1: Table S17). The DICs of the spatial lag models were smaller than the DIC of the GAM ran previously, (-3125.58 and -1744.26, respectively). Based on the ML (Maximum Likelihood), the best model was the SDEM (Eq. (9) Table 1, Additional file 1: Table S20). This was used since the DIC fitted in INLA as run by Rue et al*.* did not allow the comparison of the DICs [32]. The fit with ML and INLA, gave approximately the same values.

#### Impact

As shown in Table 2, since the indirect effect of ITNs coverage was positive and significant, therefore, for a particular cluster, increasing the ITNs coverage in its nearby clusters or day land surface in the same cluster was found to be significantly associated with the decrease of the prevalence in this cluster by 21% or 2%. An increase of wet days in a cluster or mean temperature in nearby clusters was associated with an increase of the prevalence in that cluster by 3% or 2%. There was a statistically significant difference in prevalence by type of residence.

**Table 2:** Comparison INLA and Maximum likelihood for SDEM

Covariates	SDEM from ML		SDEM from INLA—95% CI	
	Direct impact	Indirect impact	Direct impact	Indirect impact
Mean temperature	− 0.0089	0.019	− 0.009 (− 0.025; 0.0069)	0.019 (0.0012; 0.037)
Day land surface temperature	− 0.015	0.0065	− 0.015 (− 0.02; − 0.011)	0.0065 (0.0016; 0.011)
Wet days	0.032	− 0.0016	0.032 (0.012; 0.052)	− 0.0018 (− 0.023; 0.02)
ITN coverage	0.14	− 0.21	0.14 (-0.067; 0.34)	− 0.21 (− 0.4; − 0.0055)
Urban—rural	− 0.041	− 0.0083	− 0.041 (− 0.054; − 0.028)	− 0.0082 (− 0.022; 0.0052)

From M0, the spatial variation was reduced by 33%, 13% and 8% when considering the M4, the model with environmental variables (M3) and M2 respectively. Looking specifically at the ITN coverage curve, we can see that malaria prevalence only begins to fall at 20% coverage (Additional file 1: Fig. S18).

## Discussion

The aim of this work was to use Bayesian spatio-temporal analysis to identify the spatial patterns of malaria prevalence over time in Gabon for children between 2 and 10 years of age, using DHS survey data. This study considered environmental factors and other variables, such as ITNs coverage, population count, wealth index and type of residence. To grasp the complexity of malaria prevalence and the survey indicators, various model strategies were used, including one by Giorgi et al*.* [33]. Space–time smoothing model based on the areal data and the unit-level data were used for small area estimation (SAE) using the sampling design of the multi-stage stratified sampling method, which ensures the representativeness of the estimate at the national level. This study investigated the spatio-temporal distribution of malaria taking into account almost the whole area in Gabon. By incorporating these comprehensive methods, this study aimed to enhance understanding of the spatio-temporal dynamics of malaria, providing valuable insights for targeted interventions and malaria control efforts in Gabon.

The prevalence decreased between 2000 and 2010, and increased slightly after 2010. This decrease was also seen in a study investigating the changing risk of PfPR. It was conducted based on 49 African countries, and 82% of them showed this similar decrease [34]. Findings of the high prevalence in the rural areas were supported by a study conducted in Mozambique [35]. They found that children living in urban areas had lower risk of malaria compared to those living in rural zones. The decrease of malaria prevalence can be explained by the fact that new interventions were introduced around 2000 and 2006 in urban areas, for example the use of ACT and the distribution of ITNs. In fact, during this period many studies reported the introduction of these new interventions in the improvement of anti-malarial policy, and the decrease of the prevalence at the same period [2, 10]. For African countries, such as Malawi, there were some obvious decrease of the prevalence as the intervention was sustained [36].

The high prevalence in rural areas can be explained by the lack of amenities, therefore locating health care workers poses challenges in terms of availability [2]. The underlying factors responsible for the observed increasing trend, particularly the slight upturn after 2010, remain ambiguous and warrant further investigation. However, it was notice the following points [2, 5]: after 2008, maybe due to the decrease in global fund contribution or the observed decrease in different areas in Gabon, ITN coverage decreased, and the activities of National Malaria Control Programme (NMCP) for prevention slowdown. Since the interest was often focused in a particular age group, and it happened that the intervention is not sustained this led potentially to a small increase in the prevalence This is known as ‘delayed malaria’ [38]. In the studies conducted in Zambia and Zanzibar, as the intervention was maintained, the rebound, referring to a specific period characterized by an elevated risk of malaria infection following a time-limited period of protection from malaria, was not shown [36, 37]. The implication of delayed malaria and rebound in regions with inconsistent or reduced intervention efforts is a higher prevalence of malaria, as supported by many studies [2, 5]. Sustained, comprehensive intervention strategies, including consistent funding, active programme implementation, and continuous efforts in preventive measures, are crucial in combating malaria effectively and avoiding resurgence or rebound effects. This may mitigate the impact of the loss of naturally acquired immunity as it could be induced by effective interventions. Other factors able to contribute in such increase can be vector resistance to insecticide or lack of awareness campaigns. It should be notice, drug resistance poses a threat to the effectiveness of control measures. On the other hand, it will be important to arise awareness in the education system including education from parent regarding the risk of malaria and the distribution of new ITNs, to increase its average just after cessation of the effective intervention [6]. It is worthy to notice that maintaining long-term community engagement, consistent funding, and ensuring the sustainability of interventions remain challenges. The result of 46% or 41% of the contribution of spatial component in the estimation of the prevalence was in line with a study conducted in Rwanda [16], although the confidence interval was wide. The authors found that the spatial component contributed 49% in 2014 and 41% in 2018 based on malaria relative risk estimates. These results demonstrated also that it is better to use the survey design of the study and the smoothing model, since it reduced the variance due to the introduction of another random effect of borrowing strength from neighbours. This was in line with many studies in the literature [39]. Furthermore, the use of the unit-level model reduced the uncertainty in the credible interval of the estimate, while approximately the same estimate was obtained with the areal model.

The change of hotspots and cold spots over time and space was also supported by a study conducted in Senegal [40]. Here, the authors analysed the spatio-temporal distribution of hotspots and their association with various environmental variables. They found that the variation of hotspots was associated with rainfall and EVI). For this study, this relationship was further evaluated separately to explore the distribution of hotspots and its relationship with environmental and other factors in Gabon [41], and was in line with the above study. Hotspots occurrence was found to be significantly associated with the increase in year, and rainfall amount.

The difference on the significance of covariates found in the OLS regression compared to spatial models, emphasized that, in the presence of spatial autocorrelation, the results of the OLS were biased as the standard error was not correct [31]. In all spatio-temporal models used, the change in the year did not yield significant evidence of influencing the prevalence. This finding may be attributed to limited temporal observations and potential variations in data collection timing.

Among all models considered to assess the effect of covariates on the prevalence, SDEM was the best. This model was also used by Augusto et al*.* to describe the relationship between forest clear-cutting and malaria cases [42]. Using this model, it was found that environmental variables were impacting considerably the spatial distribution when they were introduced in the analysis before. This spatial distribution of the prevalence slightly changed after adjusting with the other variables. However, the magnitude of the change in the prevalence was small. This finding was in line with many other studies highlighting the importance of geo-climatic variables using other models [17, 18]. Interaction between ITNs coverage and other variables, or amongst other variables was not found. This is in contrast to other studies, where they found interaction between night land surface temperature (LSTN) and Normalized Difference Vegetation Index (NDVI) [18].

ITNs coverage was the most important variable affecting the variation of malaria prevalence in Gabon with protective effect on nearby clusters. This result was similar to the finding of a study conducted by Hawley et al*.* [43]. In their study, by investigating the effect of ITNs in the nearby households, they found that ITNs have a protective effect on households that did not receive ITNs but were situated within 300 m of the ones that did. These results from our study highlighted the important role of malaria control policy on the prevalence of malaria even on the cluster level. In the non-linear relationship when the summarized data on year were used, this study suggested that malaria prevalence started to decrease once ITNs coverage was above 20%.

The negative effect of mean temperature was consistent with many studies that did not applied spatial econometrics model [44, 45]. Despite being supported by several studies, the observed outcome was unexpected. This is due to the fact that an increase in temperature would normally be anticipated to create challenges in the life cycle of *P. falciparum*. However, the mean temperature in Gabon, according to this study was between 24 °C and 27 °C which is in the range of the optimal temperature for both vector and parasite, that is, 25 °C and 27 °C. Thus, these results were suggesting that the country has a suitable ground for mosquito breeding. The finding of the effect of day land surface temperature on the prevalence was supported by the study conducted in Uganda [18]. In their study, they found a decline of malaria at higher temperature 35 °C, but they did not apply the same model to identify the spillover effect.

The finding of the effect of wet days on malaria prevalence, was also found in a study conducted in the Democratic Republic of Congo [45]. They focused on the association of climatic factors in malaria morbidity based on data from 2001 to 2019. They found that one-day increase of the rainy day was associated with 7% increase in malaria cases. In fact, wet days have a significant positive correlation with rainfall. Rainfall leaves sites for mosquito to grow larvae, therefore contribute to expanding the mosquito population.

This study had a cross-sectional design with data collected at only four specific time points. A more comprehensive understanding of the temporal trends could be gained with more time points. The lack of some other variables like health facilities quality and proportion of drug limits the ability to fully grasp their impact on the prevalence of malaria. Also, the use of a secondary data may introduce potential temporal biases which may limit knowledge in the current situation due to changes in demographics, technology, or socio-economic factors. Although, the exact method of determining parasitaemia was not available from the data, a report of a UNAID DHS surveys carried out in 29 African countries used both rapid diagnostic test and malaria microcopy.

### Policy implication

The use of spatial component methodology allows to obtain good estimates of an outcome in small areas, reflecting the true burden of the disease, when there is the presence of a strong spatial effect. The findings of this study provide valuable insights for policy makers, enabling them to effectively target and prioritize areas with a high burden of malaria. The spatial effect observed in the level of cluster of households can be important to amplify the impact of an intervention to reach areas not targeted through the local spillover effect. ITNs coverage plays a protective role which is, in magnitude, much better than the negative spillover effect of environmental variables. Environmental variables associated with prevalence reduction and ITNs coverage can significantly improve the impact of interventions. This gives strength to the expectation that the interventions may play a significant role in the way of reducing the burden of malaria.

## Conclusions and recommendations

The findings of this study emphasize the significant prevalence of malaria, identification of hotspots, the presence of strong spatial autocorrelation decreasing slowly, and the spillover effect of environmental variables. Additionally, the study highlights the association between variables such as day land surface temperature, wet days, mean temperature, and ITNs coverage after adjusting for other factors. These insights contribute to a comprehensive understanding of the malaria landscape and inform strategies to mitigate the disease burden effectively.

These findings, may have four major implications. Firstly, the use of advanced spatial methods to achieve more accurate estimations of malaria prevalence for small areas with strong spatial autocorrelation, by incorporating spatial components in the analysis using Bayesian framework, and the spillover effect through SDEM to elucidate the impact of interventions or some factors when change occurred in surrounding neighbourhoods. Secondly, the optimization of the resource allocation. Finding suggesting that the ITN coverage may be increased at least to 20% may benefit in resource allocation. Increasing the ITNs coverage uniformly across diverse locations to a substantial threshold in different household clusters necessitates significant resources, posing challenges in achieving parity across various regions, since the limitations in resource availability often hinder attaining equal coverage levels in multiple areas. Therefore, these findings may highlight the potential cost reduction and optimization of resource allocation across various locations even in the setting where they are not enough to reach high level of the coverage. This enables more effective and efficient resource utilization to yield optimal outcomes.

Thirdly, the mitigation of hotspots. The second point coupled with enhanced healthcare access and the establishment or reinforcement of health infrastructure in specific regions, aiding in prompt diagnosis and treatment accessibility in rural areas, may fortify malaria control initiatives, significantly reducing clinical incidence, and potentially curbing the formation of hotspots. The increase in ITNs coverage paired with spillover effect at the household cluster level, may enhance the impact of the ITNs coverage on the clinical burden of malaria in close areas for children aged between 2 and 10 years. If this is not considered, the decrease of ITNs coverage, and other factors like quality of public health facilities, associated with the increase of some ecological factors, will probably lead to a significant upsurge of malaria, and formation of hotspots. Fourthly, collaborative efforts between the National Directorate of Meteorology (DGM) and the National Malaria Control Programme (NMCP) are needed to mitigate adverse climatic effects on malaria by developing a good early warning system (EWS) for malaria. Focusing research on this EWS for malaria, will help in informing population on different initiatives to undertake when there are changes in environmental or ecological factors, in order to better fight malaria at community or individual level. These recommendations should be applied particularly in targeted cluster of households in the north west, and specifically in Estuaire province and the south part of the country where there are hotspots or coldspots. Implementing these comprehensive measures can pave the way towards meaningful progress in malaria control and contribute to improving public health outcomes in the country.

### Supplementary Information

**Additional file 1.** Modelling details.

## Data Availability

The datasets analysed during the current study are available from the Demographic Health Surveys Program repository upon request, (https://dhsprogram.com/what-we-do/survey/survey-display-421.cfm).

## Appendix

See Table 3

**Table 3:** Models and related equations in estimating the prevalence of malaria and the effect of covariates

Models	Related equations	Description
Space–time smoothing estimate	(a) Prevalence estimation with weight ${p}_{it}=\frac{\sum_{k=1}^{{n}_{it}}{w}_{k}{y}_{k}}{\sum_{k=1}^{{n}_{it}}{w}_{k}}$ (1) (b) True prevalence $logit\left({p}_{it}\right)|{\lambda }_{it}\sim N\left({\lambda }_{it},{V}_{it}^{L}\right)$ ${\lambda }_{it}=\beta +{\beta }_{it}\left(rural\right)+\left(\lambda +{\gamma }_{it}\right)\left(urban\right)+{\alpha }_{it}+{\epsilon }_{it}+\frac{1}{\sqrt{\tau }}\left(\sqrt{\left(1-\phi \right)}{S}_{i}+\sqrt{\phi }{e}_{i}\right)+{\delta }_{it}$ $\phi$ is the spatial fraction(2) (c) PC Prior The prior for $\phi$ is not on a close form. The prior on $\tau$: $\pi \left(\tau \right)=\frac{\lambda }{2}{\tau }^{-3/2}{e}^{-\lambda {\tau }^{-1/2}},\tau >0,\lambda >0$ (3)	${y}_{j}$(a) was defined to be the cluster’s outcome and${w}_{k}$, if available, the weight associated with the cluster$k$. $i\in I$, $I$ is the partition of the region, and ${n}_{it}$ is the number of clusters at time $t$ in the area$i$ (b) the true prevalence was estimated using $logit\left({p}_{it}\right)$.$\beta$ is the intercept for a cluster identified as in rural area, $\beta +\lambda$ intercept for urban area. ${\beta }_{it}$ and ${\gamma }_{it}$ are temporal main time-varying effects. ${\alpha }_{it}$ is the structured time trends, ${\epsilon }_{it}$ is the temporal term ($iid$ XE “${\text{iid}}$”), ${S}_{i}$is the structured spatial trends, ${e}_{i}$ the unstructured spatial terms and ${\delta }_{it}$ the interaction between space and time (c) The priors were defined on the hyperparameters $\phi$ and $\tau$ for the for the structured and instructured effect [26] For time effect $\alpha$, the following was used: ${\alpha }_{it}\sim N\left({\alpha }_{t-1},{\sigma }^{2}\right)$ (random walk order 1) or $N\left(2{\alpha }_{t-1}+{\alpha }_{t-2},{\sigma }^{2}\right)$ (random walk order 2). The auto-regressive model AR(1) is defined as ${\alpha }_{t}=\phi {\alpha }_{t-1}+{\omega }_{t} {\omega }_{t}\sim N\left(0,{k}^{\left(-1\right)}\right)$, $t\ge 2$ and ${\alpha }_{1}\sim N\left(0,{\tau }_{1}\right)$, ${\tau }_{1}=k\left(1-{\phi }^{2}\right)$. The spatial structure of ${\delta }_{it}$ is modelled by the ICAR model: ${\delta }_{it}|{\delta }_{jt},{\tau }_{\delta }\sim N\left(\frac{1}{{n}_{i}}\sum_{j\in {n}_{\partial i}}{\delta }_{jt},\frac{{t}_{\delta }}{{n}_{i}}\right)$, $\partial i$= the set of neighbours for the area $i$, ${n}_{\partial i}$ number of these neighbours
SPTOLS	${y}_{it}\sim \mathcal{N}\left({\eta }_{it},{\sigma }_{e}^{2}\right)$ ${\eta }_{it}=\sum_{j=1}^{p}{\beta }_{j}{x}_{ji}+{\omega }_{it}, {\omega }_{it}=a{\omega }_{i\left(t-1\right)}+{\xi }_{it}, \left|a\right|<1$(4)	${x}_{mi}$ is the mth variable values at cluster $i$, ${\sigma }_{e}^{2}$ the variance for the measurement error modelled through a Gaussian process spatially and serially uncorrelated. Here ${\omega }_{it}$ is an AR(1). Coefficient $a$ is spatially correlated. ${\xi }_{it}$ is a GF (Gaussian Field) independent in time, with mean $0$ such that for ${t}_{1}={t}_{2}$, $Cov\left({\xi }_{i{t}_{1}},{\xi }_{i{t}_{2}}\right)$ is non-null and is the Matèrn covariance function defined as above. The PC prior was used for the autocorrelation parameter $a$ such that the probability of the standard deviation to be greater than 0 is 0.9. For the remaining parameters, loggamma distributions were used with mean 1, and precision of 0.00001 or 0.001 as also used by Musenge et al. [25]
GAM	${y}_{it}\sim \mathcal{N}\left({\eta }_{it},{\sigma }_{e}^{2}\right)$ ${\eta }_{it}=\sum_{j=1}^{p}{f}_{j}\left({x}_{ji}\right)+{v}_{it}, {v}_{it}=\rho {v}_{i\left(t-1\right)}+{u}_{it}$ • Space time correlated GAM: $\rho$ is estimated Replicated model: $\rho$ = 0 (5)	The prior specification for the functions ${U}_{j}$ is ${f}_{j}$ for all $j$ is a random walk process of order 2 (RW(2)). $f\left(t\right)=2f\left(t-1\right)-f\left(t-2\right)+u\left(t\right)$, $f\left(t\right)=f\left({x}_{\left(t\right)}\right)$, ${x}_{\left(t\right)}$ is an ordered statistic, with $u\left(t\right)\sim N\left(0,{\tau }^{2}\right)$ and diffuse prior set on $f\left(1\right)$ and $f\left(2\right)$
Spatial econometrics	$y=X\beta +u$; with $u=\rho Mu+e$ with $e\sim MVN\left(0,{\sigma }^{2}{I}_{n}\right)$ (6) SEM, lag on the error: The average total impact (direct) is the posterior marginal of the coefficient ${\beta }_{r}$ $y=\rho Wy+X\beta +e$;$e\sim MVN\left(0,{\sigma }^{2}{I}_{n}\right)$ (7) The SLM, $y=\rho Wy+X\beta +WX\gamma +e$; $e\sim MVN\left(0,{\sigma }^{2}{I}_{n}\right)$(8) The SDM: The average impact: ${n}^{-1}tr\left({\left({I}_{n}+\rho W\right)}^{-1}\right){\beta }_{r}+{n}^{-1}tr\left({\left({I}_{n}+\rho W\right)}^{-1}W\right){\gamma }_{r})$, $y=X\beta +WX\gamma +u$; $u=\rho Wu+e$, $e\sim MVN\left(0,{\sigma }^{2}{I}_{n}\right)$(9) SDEM: The average total impact: ${\beta }_{r}+{\gamma }_{r}$	These are implemented in INLA as spatial latent model by conditioning on $\rho$ to allow the model to take the suitable form (53). In this estimation, a Gaussian prior was used for $log\left(\frac{\rho }{1-\rho }\right)$, and a normal prior for $\beta$ with mean $0$ and very large variance In the implementation, the variance of the likelihood was set to ${e}^{-15}$ [32]. Therefore, it is not possible to compare models fitted within INLA, but the obtained DIC can perhaps be compared with the one obtained with the other models such as the GAM
SPDE	${\left({k}^{2}-\Delta \right)}^{\alpha /2}\left(\tau f\left(x\right)\right)=W\left(x\right)$ with $cov\left(f\left(0\right),f\left(x\right)\right)=\frac{{\sigma }^{2}}{{2}^{v-1}\Gamma \left(v\right)}{\left(k\parallel x\parallel \right)}^{v}{K}_{v}\left(k\parallel x\| \right)$ (10) f is a weak solution	W(x) is a Gaussian white nose process, $\Delta$ is the Laplacian, $k$ is a s cale parameter related to the range $r$, $\tau$ affects the variance of $f$, $\alpha$ is related to the smoothness of $f$. This solution is approximated by a GMRF through triangulation The range is the distance after which the spatial correlation is small, i.e. almost null or less than 0.1. The default value for $\alpha$ was $2$. Specification on the range is provided in Supplementary material $r=\frac{\sqrt{8}}{k}$

## References

[1] World malaria report 2021. [cited 2022 Sep 16]. https://www.who.int/teams/global-malaria-programme/reports/world-malaria-report-2021

[2] Mawili-Mboumba DP, Akotet MKB, Kendjo E, Nzamba J, Medang MO, Mbina J-RM (2013). Increase in malaria prevalence and age of at risk population in different areas of Gabon. Malar J.

[3] Sustainable Development Report 2022. [cited 2023 Jun 16]. https://dashboards.sdgindex.org/

[4] Evaluation of direct costs associated with the management of clinical stage of malaria in children under five years old in Gabon—ProQuest. [cited 2023 Jun 30]. https://www.proquest.com/openview/bbe97de5bba650f04b19dbb2f271ac3b/1?pq-origsite=gscholar&cbl=42600 10.1186/s12936-021-03862-4 PMC8325277 34330288

[5] Moukandja IP, Essone JCBB, Sagara I, Kassa RFK, Ondzaga J, Douki J-BL (2016). Marked rise in the prevalence of asymptomatic plasmodium falciparum infection in rural Gabon. PLOS ONE.

[6] Lendongo-Wombo J-B, Oyegue-Liabagui S-L, Biteghe-Bi-Essone J-C, Ngoungou EB, Lekana-Douki J-B (2022). Epidémiology of malaria from 2019 to 2021 in the southeastern city of Franceville. Gabon BMC Public Health.

[7] Bejon P, Warimwe G, Mackintosh CL, Mackinnon MJ, Kinyanjui SM, Musyoki JN (2009). Analysis of immunity to febrile malaria in children that distinguishes immunity from lack of exposure. Infect Immun.

[8] Smith DL, Guerra CA, Snow RW, Hay SI (2007). Standardizing estimates of the Plasmodium falciparum parasite rate. Malar J.

[9] Patil AP, Okiro EA, Gething PW, Guerra CA, Sharma SK, Snow RW (2009). Defining the relationship between Plasmodium falciparum parasite rate and clinical disease: statistical models for disease burden estimation. Malar J.

[10] Bouyou-Akotet MK, Mawili-Mboumba DP, Kendjo E, Mabika-Mamfoumbi M, Ngoungou EB, Dzeing-Ella A (2009). Evidence of decline of malaria in the general hospital of Libreville, Gabon from 2000 to 2008. Malar J.

[11] Assele V, Ndoh GE, Nkoghe D, Fandeur T (2015). No evidence of decline in malaria burden from 2006 to 2013 in a rural province of Gabon: implications for public health policy. BMC Public Health.

[12] Maghendji-Nzondo S, Nzoughe H, Lemamy GJ, Kouna LC, Pegha-Moukandja I, Lekoulou F (2016). Prevalence of malaria, prevention measures, and main clinical features in febrile children admitted to the Franceville Regional Hospital, Gabon. Parasite.

[13] Craig MH, Snow RW, le Sueur D (1999). A climate-based distribution model of malaria transmission in sub-Saharan Africa. Parasitol Today.

[14] Mordecai EA, Paaijmans KP, Johnson LR, Balzer C, Ben-Horin T, de Moor E (2013). Optimal temperature for malaria transmission is dramatically lower than previously predicted. Ecol Lett.

[15] Ndlovu N. Analysis of the geographical patterns of malaria transmission in KwaZulu-Natal, South Africa using Bayesian Spatio-temporal modelling. 115.

[16] Semakula M, Niragire F, Faes C (2020). Bayesian spatio-temporal modeling of malaria risk in Rwanda. PLoS ONE.

[17] Nkurunziza H, Gebhardt A, Pilz J (2010). Bayesian modelling of the effect of climate on malaria in Burundi. Malar J.

[18] Ssempiira J, Kissa J, Nambuusi B, Mukooyo E, Opigo J, Makumbi F (2018). Interactions between climatic changes and intervention effects on malaria spatio-temporal dynamics in Uganda. Parasite Epidemiol Control.

[19] DHS Covariate Extraction [Internet]. The DHS Program; 2022 [cited 2022 Aug 24]. https://github.com/DHSProgram/DHS-covariate-extraction

[20] Seyoum D, Yewhalaw D, Duchateau L, Brandt P, Rosas-Aguirre A, Speybroeck N (2017). Household level spatio-temporal analysis of Plasmodium falciparum and Plasmodium vivax malaria in Ethiopia. Parasit Vectors.

[21] Elkasabi M, Ren R, Pullum T. Multilevel Modeling Using DHS Surveys: a Framework to Approximate Level-Weights. 2020.

[22] Mayala B, Fish TD, Eitelberg D, Dontamsetti T. The DHS Program Geospatial Covariate Datasets Manual. The Demographic and Health Surveys Programme. 2^nd^ Edn. ICF, Rockville, USA. 2018. https://fdocuments.net/document/the-geospatial-covariate-datasets-manual-2020-03-17-1-the-dhs-program-geospatial.html

[23] Rue H, Martino S, Lindgren F, Simpson D, Riebler A, Krainski E. INLA: Functions Which Allow to Perform Full Bayesian Analysis of Latent Gaussian Models Using Integrated Nested Laplace Approximaxion. R package version 0.0–1389624686. 2014.

[24] Rue H, Martino S, Chopin N (2009). Approximate Bayesian inference for latent Gaussian models by using integrated nested Laplace approximations. J Royal Stat Soc Series B.

[25] Musenge E, Chirwa TF, Kahn K, Vounatsou P (2013). Bayesian analysis of zero inflated spatiotemporal HIV/TB child mortality data through the INLA and SPDE approaches: Applied to data observed between 1992 and 2010 in rural North East South Africa. Int J Appl Earth Obs Geoinf.

[26] Simpson DP, Rue H, Martins TG, Riebler A, Sørbye SH. Penalising model component complexity: A principled, practical approach to constructing priors. arXiv; 2015 Aug. Report No.: arXiv:1403.4630. http://arxiv.org/abs/1403.4630

[27] Moraga P. Chapter 9 Spatial modeling of geostatistical data. Malaria in The Gambia | Geospatial Health Data: Modeling and Visualization with R-INLA and Shiny. [cited 2022 Jun 13]. https://www.paulamoraga.com/book-geospatial/sec-geostatisticaldataexamplespatial.html

[28] Miller DL, Glennie R, Seaton AE (2020). Understanding the stochastic partial differential equation approach to smoothing. JABES.

[29] Bivand R, Gómez-Rubio V, Rue H (2015). Spatial data analysis with R-INLA with some extensions. J Stat Softw.

[30] Anselin L. Spatial Econometrics: Methods and Models. Springer Science & Business Media; 1988.

[31] Duque Y. LeSage & Pace (2009) Introduction to Spatial Econometrics Statistics A Series of Textbooks and Monographs. 2020.

[32] Gomez-Rubio V, Bivand RS, Rue H. Estimating Spatial Econometrics Models with Integrated Nested Laplace Approximation. arXiv; 2021 May. Report No.: arXiv:1703.01273. http://arxiv.org/abs/1703.01273

[33] Giorgi E, Fronterrè C, Macharia PM, Alegana VA, Snow RW, Diggle PJ (2021). Model building and assessment of the impact of covariates for disease prevalence mapping in low-resource settings: to explain and to predict. J Royal Soc Int.

[34] Noor AM, Kinyoki DK, Mundia CW, Kabaria CW, Mutua JW, Alegana VA (2014). The changing risk of Plasmodium falciparum malaria infection in Africa: 2000–10: a spatial and temporal analysis of transmission intensity. The Lancet.

[35] Ejigu BA (2020). Geostatistical analysis and mapping of malaria risk in children of Mozambique. PLoS ONE.

[36] Spence-Lewis IM (2011). Scaling up malaria control in Zambia: progress and impact 2005–2008. Am J Trop Med Hyg.

[37] Beer N, Ali AS, Shakely D, Elfving K, Al-Mafazy A-WH, Msellem M (2013). High effective coverage of vector control interventions in children after achieving low malaria transmission in Zanzibar, Tanzania. Malar J.

[38] Greenwood B, Zongo I, Dicko A, Chandramohan D, Snow RW, Ockenhouse C (2022). Resurgent and delayed malaria. Malar J.

[39] Mercer L, Wakefield J, Chen C, Lumley T (2014). A comparison of spatial smoothing methods for small area estimation with sampling weights. Spat Stat.

[40] Dieng S, Ba EH, Cissé B, Sallah K, Guindo A, Ouedraogo B (2020). Spatio-temporal variation of malaria hotspots in Central Senegal, 2008–2012. BMC Infect Dis.

[41] Mougeni F, Lell B, Ngianga K, Chirwa T. PA-769 Bayesian spatio-temporal analysis of malaria hotspot in Gabon from 2000 to 2015. BMJ Global Health [Internet]. 2023 [cited 2023 Dec 21];8. https://gh.bmj.com/content/8/Suppl_10/A120.3

[42] Santos AS, Almeida AN (2018). The impact of deforestation on malaria infections in the Brazilian Amazon. Ecol Econ.

[43] Hawley WA, Phillips-Howard PA, ter Kuile FO, Terlouw DJ, Vulule JM, Ombok M (2003). Community-wide effects of permethrin-treated bed nets on child mortality and malaria morbidity in western Kenya. Am J Trop Med Hyg.

[44] Mohammadkhani M, Khanjani N, Bakhtiari B, Tabatabai SM, Sheikhzadeh K (2019). The relation between climatic factors and malaria incidence in Sistan and Baluchestan. Iran SAGE Open.

[45] Panzi EK, Okenge LN, Kabali EH, Tshimungu F, Dilu AK, Mulangu F (2022). Geo-climatic factors of malaria morbidity in the democratic Republic of Congo from 2001 to 2019. Int J Environ Res Public Health.

