# Supplementary material for: Bayesian spatio-temporal analysis of malaria prevalence in children between 2 and 10 years of age in Gabon
Source: Malar J. 2024 Feb 23;23:57. doi: 10.1186/s12936-024-04880-8 (PMC10893641; doi:10.1186/s12936-024-04880-8)
Supplement: Supplementary file 1 — Additional file 1. Modelling details. [file 12936_2024_4880_MOESM1_ESM.pdf]

# Supplementary document

## 1.1 Descriptive analysis

### 1.1.1 Distribution of the prevalence and the covariates

Before the estimation of the prevalence, 64 clusters were removed because of missing values, 20 clusters for the variable malaria prevalence and 44 clusters in Libreville and Port-Gentil. These were removed because many values for these towns were missing. These were identified in DHS database as -9.999 (showing Not Available [NA]), including those for which weight was not found. Only 272 remained for areal data analysis. These 272 were only for the estimation of the prevalence. For the other analysis, 316 clusters were considered.

***Table 1**: Distribution of malaria prevalence by type of residence and year*

| Province | Type of residence | Mean prevalence (SD) % | | | |
|---|---|---|---|---|---|
| | | 2000 | 2005 | 2010 | 2015 |
| Estuaire | R | 0.54 (0.03) | 0.18 (0.05) | 0.17 (0.03) | 0.32 (0.02) |
| | U | 0.48 (0.07) | 0.12 (0.03) | 0.13 (0.03) | 0.27 (0.04) |
| | | | | | |
| Haut-Ogooue | R | 0.35 (0.07) | 0.22 (0.1) | 0.14 (0.06) | 0.19 (0.05) |
| | U | 0.26 (0.04) | 0.12 (0.04) | 0.08 (0.02) | 0.13 (0.02) |
| | | | | | |
| Moyen-Ogooue | R | 0.45 (0.06) | 0.16 (0.06) | 0.12 (0.03) | 0.27 (0.03) |
| | U | 0.36 (0.04) | 0.11 (0.02) | 0.08 (0.01) | 0.2 (0.03) |
| | | | | | |
| Ngounie | R | 0.4 (0.03) | 0.11 (0.04) | 0.13 (0.02) | 0.24 (0.03) |
| | U | 0.31 (0.04) | 0.07 (0.02) | 0.09 (0.01) | 0.18 (0.03) |
| | | | | | |
| Nyanga | R | 0.3 (0.04) | 0.2 (0.06) | 0.14 (0.02) | 0.2 (0.03) |
| | U | 0.25 (0.04) | 0.13 (0.04) | 0.1 (0.02) | 0.15 (0.03) |
| | | | | | |
| Ogooue-Ivindo | R | 0.4 (0.05) | 0.12 (0.04) | 0.12 (0.02) | 0.26 (0.04) |
| | U | 0.29 (0.04) | 0.07 (0.03) | 0.08 (0.01) | 0.17 (0.04) |
| | | | | | |
| Ogooue-Lolo | R | 0.39 (0.02) | 0.11 (0.04) | 0.12 (0.01) | 0.22 (0.02) |

| | | | | | |
|---|---|---|---|---|---|
| | U | 0.27 (0.03) | 0.05 (0.01) | 0.07 (0.01) | 0.14 (0.02) |
| Ogooue Maritime | R | 0.39 (0.04) | 0.14 (0.03) | 0.07 (0.01) | 0.23 (0.02) |
| | U | 0.25 (0.04) | 0.11 (0) | 0.05 (0) | 0.14 (0.01) |
| Woleu-Ntem | R | 0.36 (0.06) | 0.2 (0.09) | 0.19 (0.05) | 0.27 (0.03) |
| | U | 0.23 (0.08) | 0.08 (0.03) | 0.12 (0.02) | 0.19 (0.04) |

***Table 2**: Covariates distribution by year and type of residence*

| **Province** | **Year** | **Prevalence Mean (SD)** | **Population count Mean (SD)** | **ITN coverage Mean (SD)** | **Aridity Mean (SD)** | **Rainfall (mm) Mean (SD)** | **Wet days (day(s)) Mean (SD)** | **EVI Mean (SD)** | **Day Land Surface Temp (°C) Mean (SD)** |
|---|---|---|---|---|---|---|---|---|---|
| Estuaire | 2000 | 0.52 (0.06) | | | 76.03 (1.39) | 2673 (2610) | 14.96 (0.43) | 320.6 (48.7) | 26.74 (1.33) |
| | 2005 | 0.16 (0.05) | 114700 (186000) | 0.16 (0.03) | 55.68 (1.07) | 2209 (1260) | 12.22 (0.36) | 343.7 (54.9) | 26.86 (1.2) |
| | 2010 | 0.16 (0.03) | 128400 (208100) | 0.34 (0.06) | 63.41 (0.99) | 2457 (1780) | 13.09 (0.41) | 344.4 (54.6) | 27.54 (1.51) |
| | 2015 | 0.3 (0.04) | 143600 (232900) | 0.06 (0.01) | 48.35 (0.77) | 1985 (1410) | 11.61 (0.36) | 347.6 (55.8) | 27.24 (1.12) |
| Haut-Ogooue | 2000 | 0.29 (0.07) | | | 56.21 (2.68) | 2189 (1490) | 11.97 (0.36) | 386.8 (34.3) | 28.07 (1.73) |
| | 2005 | 0.15 (0.08) | 11800 (13300) | 0.17 (0.02) | 50.98 (1.99) | 1715 (85) | 11.59 (0.34) | 410 (42.6) | 27.66 (1.75) |
| | 2010 | 0.1 (0.04) | 13200 (14900) | 0.38 (0.04) | 58.48 (2.38) | 1894 (120) | 12.15 (0.33) | 409.7 (42.7) | 27.91 (1.61) |
| | 2015 | 0.15 (0.04) | 14800 (16700) | 0.06 (0.02) | 51.88 (2.16) | 1620 (72) | 11.12 (0.31) | 409.9 (42.2) | 28.16 (1.68) |
| Moyen-Ogooue | 2000 | 0.41 (0.07) | | | 72.13 (1.94) | 1929 (130) | 13.15 (0.23) | 370.2 (25.5) | 25.4 (0.37) |
| | 2005 | 0.14 (0.05) | 4200 (5400) | 0.18 (0.03) | 51.88 (1.03) | 1719 (103) | 10.73 (0.22) | 391.5 (25.3) | 25.2 (0.43) |
| | 2010 | 0.11 (0.03) | 4700 (6000) | 0.41 (0.04) | 61.57 (1.33) | 1889 (134) | 11.54 (0.19) | 401.5 (31.3) | 25.36 (0.71) |
| | 2015 | 0.24 (0.04) | 5300 (6800) | 0.07 (0.04) | 46.46 (1.17) | 1465 (92) | 10.13 (0.21) | 398.2 (29.6) | 25.32 (0.53) |
| Ngounie | 2000 | 0.36 (0.06) | | | 74 (5.08) | 2326 (160) | 13.2 (0.49) | 365.4 (22.6) | 26.41 (1.4) |
| | 2005 | 0.09 (0.04) | 1900 (2200) | 0.17 (0.03) | 57.5 (3.14) | 1837 (87) | 11.35 (0.33) | 386.7 (30.6) | 25.91 (1.23) |
| | 2010 | 0.11 (0.03) | 2100 (2500) | 0.4 (0.04) | 68.91 (3.78) | 2174 (96) | 12.33 (0.42) | 386.1 (29.3) | 26.69 (1.5) |

| | | | | | | | | | |
|---|---|---|---|---|---|---|---|---|---|
| | 2015 | 0.21 (0.04) | 2300 (2800) | 0.07 (0.03) | 55.85 (2.4) | 1561 (109) | 10.76 (0.3) | 387.6 (31.6) | 26.7 (1.56) |
| Nyanga | 2000 | 0.28 (0.05) | | | 60.96 (5.44) | 1753 (228) | 11.64 (0.59) | 355.7 (49.7) | 27.07 (1.28) |
| | 2005 | 0.16 (0.07) | 1200 (1000) | 0.19 (0.02) | 48.53 (3.41) | 1397 (164) | 10.25 (0.38) | 369.4 (45.7) | 26.73 (1.37) |
| | 2010 | 0.12 (0.03) | 1300 (1200) | 0.41 (0.03) | 59.58 (3.64) | 1638 (214) | 11.13 (0.43) | 381.2 (47.9) | 26.91 (1.27) |
| | 2015 | 0.18 (0.04) | 1500 (1300) | 0.09 (0.03) | 50.64 (2.16) | 1169 (107) | 9.67 (0.49) | 382.7 (47) | 26.65 (1.16) |
| Ogooue-Ivindo | 2000 | 0.36 (0.07) | | | 54.34 (4.57) | 1701 (116) | 11.14 (0.45) | 400.1 (34.2) | 25.59 (0.73) |
| | 2005 | 0.1 (0.04) | 2400 (3600) | 0.24 (0.04) | 47.74 (1.31) | 1459 (75) | 10.23 (0.13) | 431.4 (43.9) | 25.32 (0.6) |
| | 2010 | 0.1 (0.02) | 2600 (4000) | 0.52 (0.02) | 53.74 (1.4) | 1503 (52) | 11.07 (0.13) | 430.7 (37.7) | 25.56 (0.71) |
| | 2015 | 0.22 (0.06) | 3000 (4500) | 0.12 (0.03) | 43.98 (1.69) | 1350 (90) | 9.56 (0.19) | 432.4 (40.6) | 25.67 (0.66) |
| Ogooue-Lolo | 2000 | 0.34 (0.07) | | | 61.41 (5.55) | 1921 (188) | 11.75 (0.71) | 400.7 (17.3) | 25.65 (0.75) |
| | 2005 | 0.09 (0.04) | 3100 (3700) | 0.18 (0.05) | 52.64 (2.91) | 1503 (165) | 10.77 (0.5) | 432.1 (14.8) | 25.41 (0.67) |
| | 2010 | 0.1 (0.03) | 3400 (4100) | 0.42 (0.06) | 61.19 (3.43) | 1750 (169) | 11.54 (0.54) | 445.8 (16.4) | 26.01 (0.93) |
| | 2015 | 0.19 (0.05) | 3900 (4600) | 0.07 (0.05) | 50.48 (2.75) | 1411 (136) | 10.18 (0.48) | 446.9 (16.1) | 25.94 (0.93) |
| Ogooue Maritime | 2000 | 0.34 (0.08) | | | 67.75 (1.52) | 2018 (200) | 12.31 (0.33) | 279.8 (86.4) | 26.07 (0.65) |
| | 2005 | 0.13 (0.03) | 500 (1600) | 0.27 (0.04) | 49.41 (2.75) | 1668 (192) | 10.25 (0.17) | 311.1 (96.6) | 25.98 (0.69) |
| | 2010 | 0.06 (0.02) | 600 (1800) | 0.51 (0.07) | 60.33 (3.68) | 1971 (189) | 10.89 (0.27) | 309.1 (92.1) | 26.17 (0.66) |
| | 2015 | 0.19 (0.05) | 600 (2000) | 0.18 (0.05) | 49.99 (1.99) | 1526 (243) | 9.82 (0.34) | 309.7 (91.7) | 25.97 (0.62) |
| Woleu-Ntem | 2000 | 0.31 (0.09) | | | 56.1 (5.3) | 1654 (135) | 12.33 (0.65) | 398.6 (21.3) | 25.03 (0.39) |
| | 2005 | 0.15 (0.09) | 5900 (7400) | 0.19 (0.03) | 47.48 (2.3) | 2673 (2610) | 11.14 (0.39) | 425.2 (22.9) | 24.84 (0.51) |
| | 2010 | 0.16 (0.05) | 6600 (8200) | 0.42 (0.05) | 52.63 (2.4) | 2209 (1260) | 11.97 (0.52) | 427.3 (29.1) | 25.04 (0.45) |
| | 2015 | 0.24 (0.05) | 7300 (9200) | 0.08 (0.07) | 40.74 (2.97) | 2457 (1780) | 9.97 (0.39) | 427.6 (27) | 24.92 (0.54) |

### 1.1.2 Visualization of malaria prevalence

As shown in Figure 1 *(see appendix)*, the distribution of the prevalence was right skewed. To reduce the skewness and to approximate by the normal distribution, the prevalence was transformed using the log or the square root.

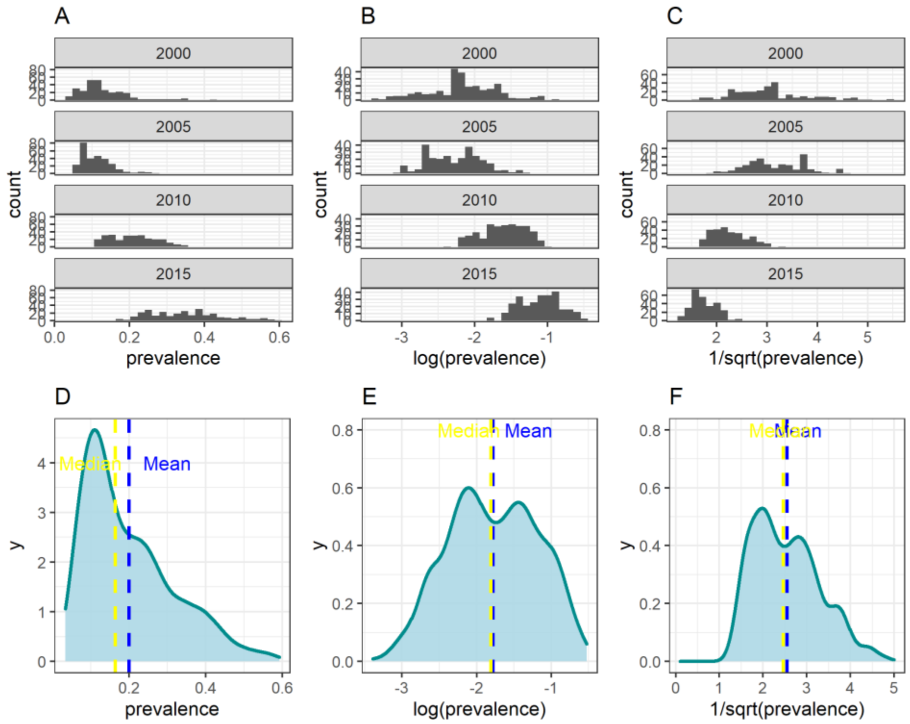


**Figure 1**: Panel (A), (B) and (C) show the distribution of the prevalence using histogram before and after transforming with log and square root for each year. Panel (D), (E) and (F) show the density in overall for each transformation

After the transformations, no one of the other transformations (Figure 2) was following the normal distribution even by type of residence.

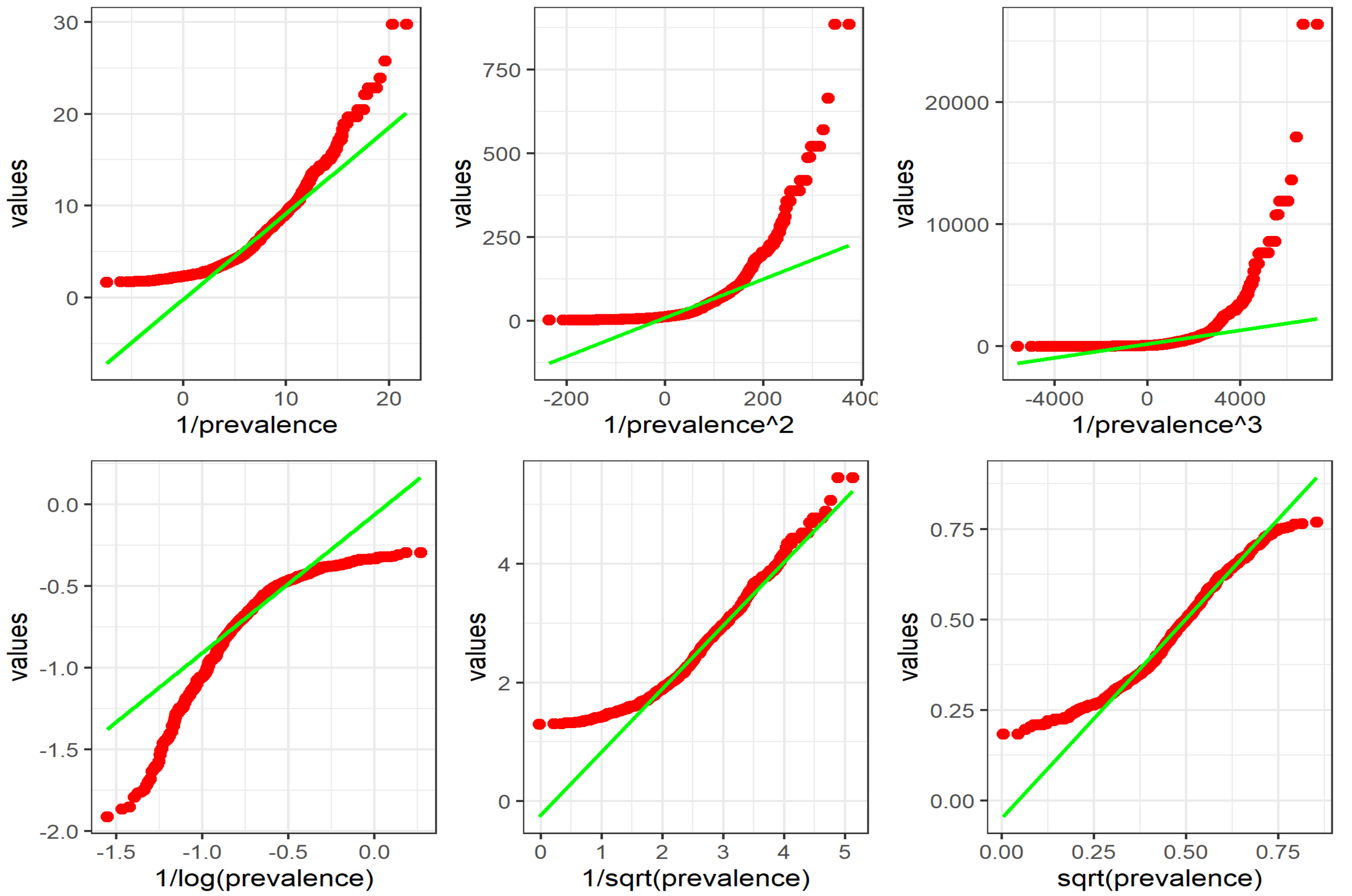


***Figure 2**: Overview of transformation of the prevalence using normal plot probability to approximate normal distribution*

However, as shown in Figure 3, the log transformation helped to obtain a more symmetric distribution but bimodal. From the normal probability plot, some departures from the ideal line at the start and the end was observed. For the inverse square root transformation, the distribution was also bimodal, somehow symmetric but with tail. From the normal probability plot, there was some departures from the ideal line only at the starting point. In contrast to the log transformation, the mean and median were slightly different for the inverse transformation (0.08 vs 0.02). Therefore, more symmetric with the log transformation than the inverse square root

transformation. Hence, normality was assumed or even the theorem of central limit (TCL) was used to assume normality even for this bimodal distribution.

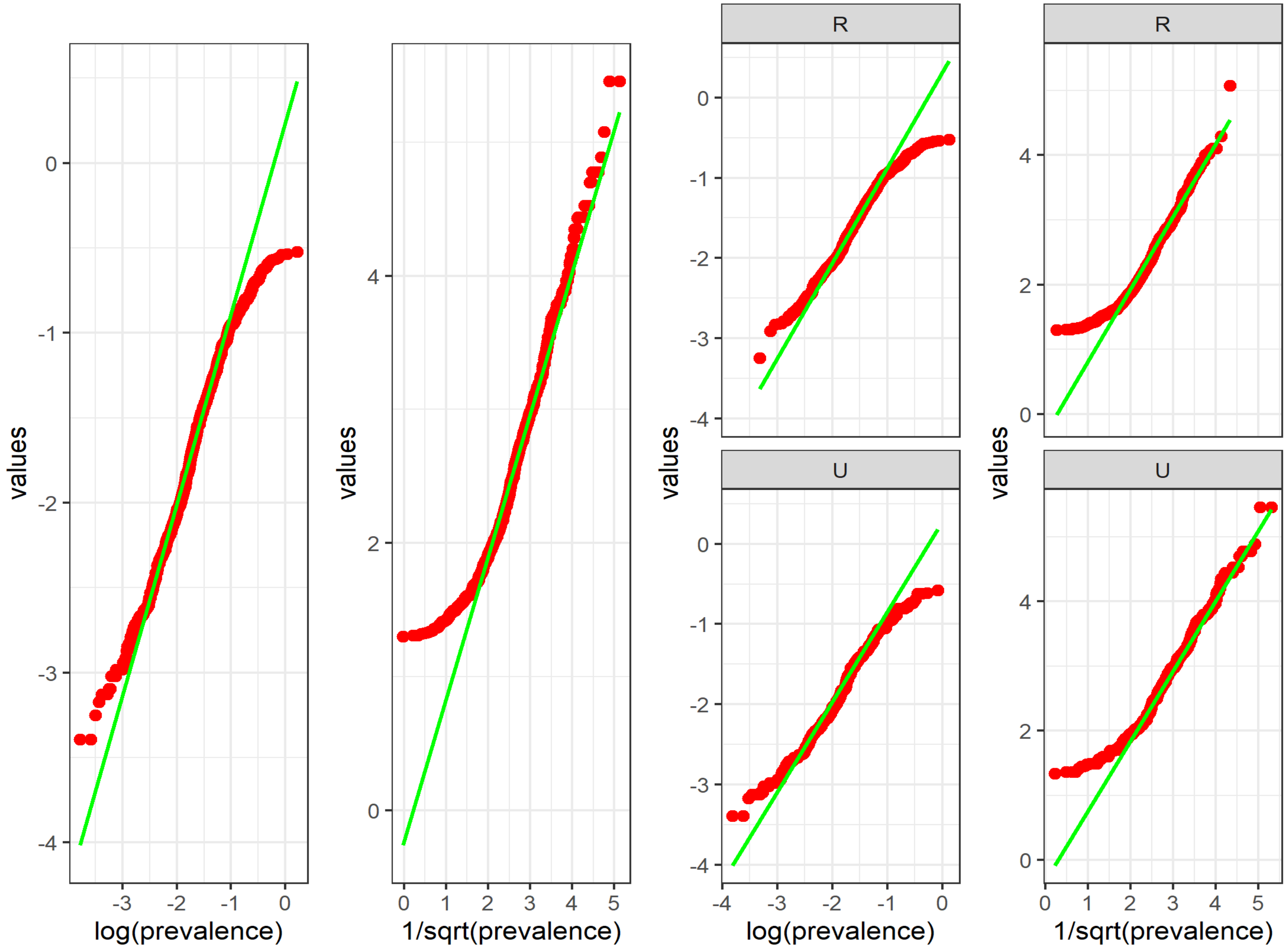


***Figure 3****: Distribution of the prevalence using normal plot probability to compare log and inverse square root prevalence in overall (left) and by type of residence (Urban-Rural - right)*

The same was found for the distribution by type of residence (Figure 4).

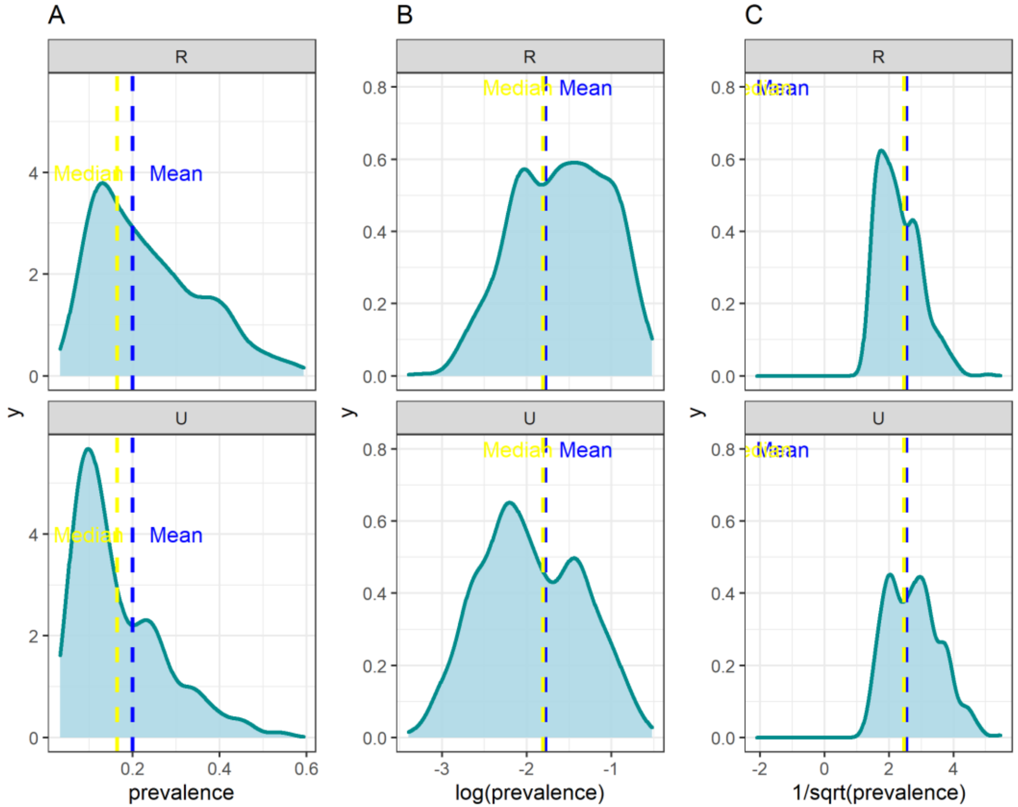


***Figure 4***: *Panel (A), (B) and (C) show the distribution of the prevalence using histogram before transforming and after transforming with log and square root Prevalence in overall by type of residence (Urban-Rural)*

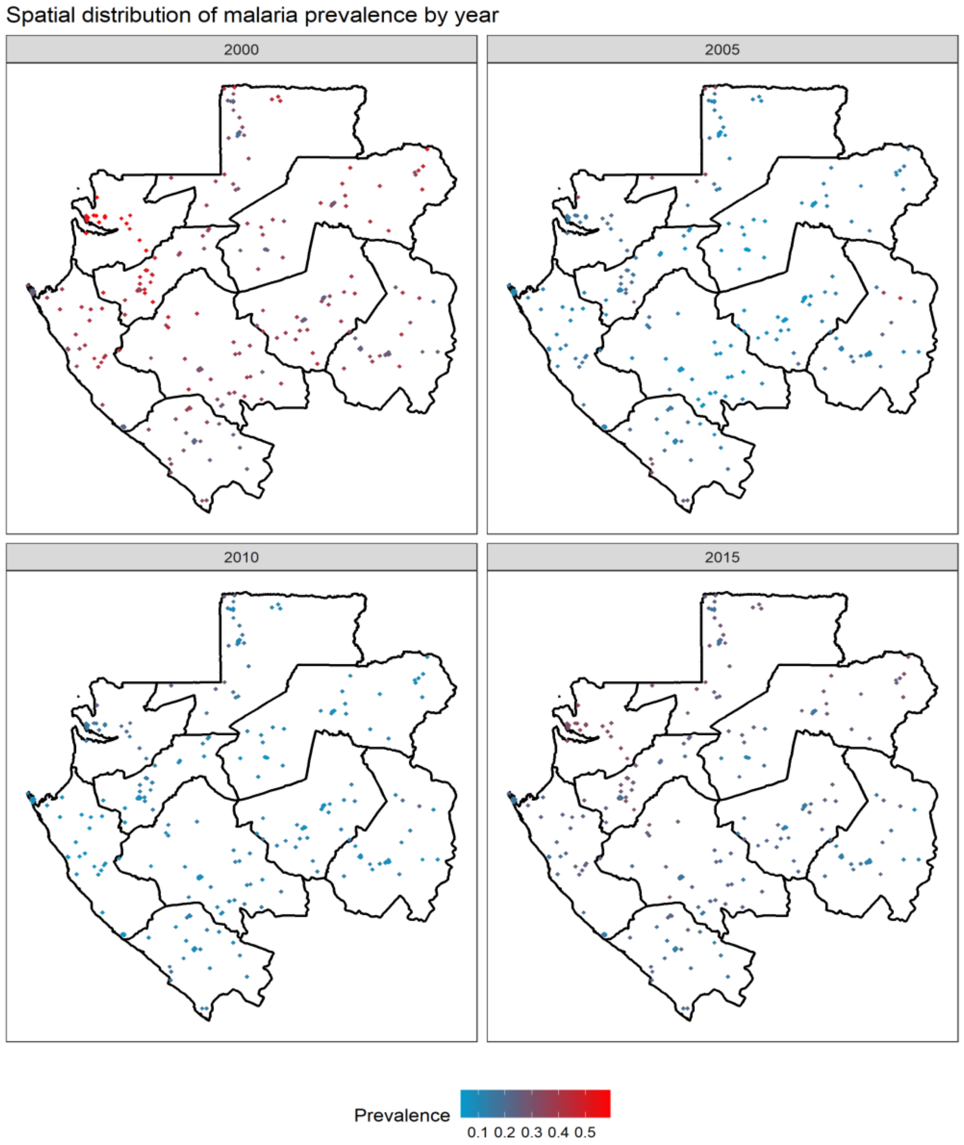


***Figure 5***: *Map of prevalence by cluster and over time in all Gabon before estimating with smooth and complexity of the survey*

### 1.1.1 Space-time estimation of the prevalence

***Table 3***: *Comparison of models based DIC for interaction effect*

| Random Walk type | Space-time type | DIC | Interaction |
|---|---|---|---|
| rw1 | 0 | -143.4477 | Model without interaction |
| rw2 | 0 | -141.2145 | Model without interaction |
| rw1 | 1 | -144.8954 | Time and space iid |
| rw2 | 1 | -145.1627 | Time and space iid |
| rw1 | 2 | -145.6341 | Temporal trends in each cluster are not spatially correlated |
| rw2 | 2 | -145.8665 | Temporal trends in each cluster are not spatially correlated |
| rw1 | 3 | -140.3611 | Time-specific spatial patterns are not temporally correlated |
| rw2 | 3 | -142.4178 | Time-specific spatial patterns are not temporally correlated |
| rw1 | 4 | -143.4477 | Cluster-specific temporal trends spatially correlated - or spatial patterns temporally correlated |
| rw2 | 4 | -141.2145 | Cluster-specific temporal trends spatially correlated - or spatial patterns temporally correlated |

rw = random walk, DIC = Deviance Information Criterion

***Table 4**: DIC model interaction vs without interaction*

| Model | DIC | Spatial fraction (SD)[a] | 95% CI[b] |
|---|---|---|---|
| Model 0 | -141.2145 | 0.35 (0.27) | 0.02 - 0.91 |
| Model 1 | -145.8665 | 0.41 (0.29) | 0.02 - 0.95 |

[a]Spatial fraction is the mixing parameter in the model;

[b] CI = Credible Interval

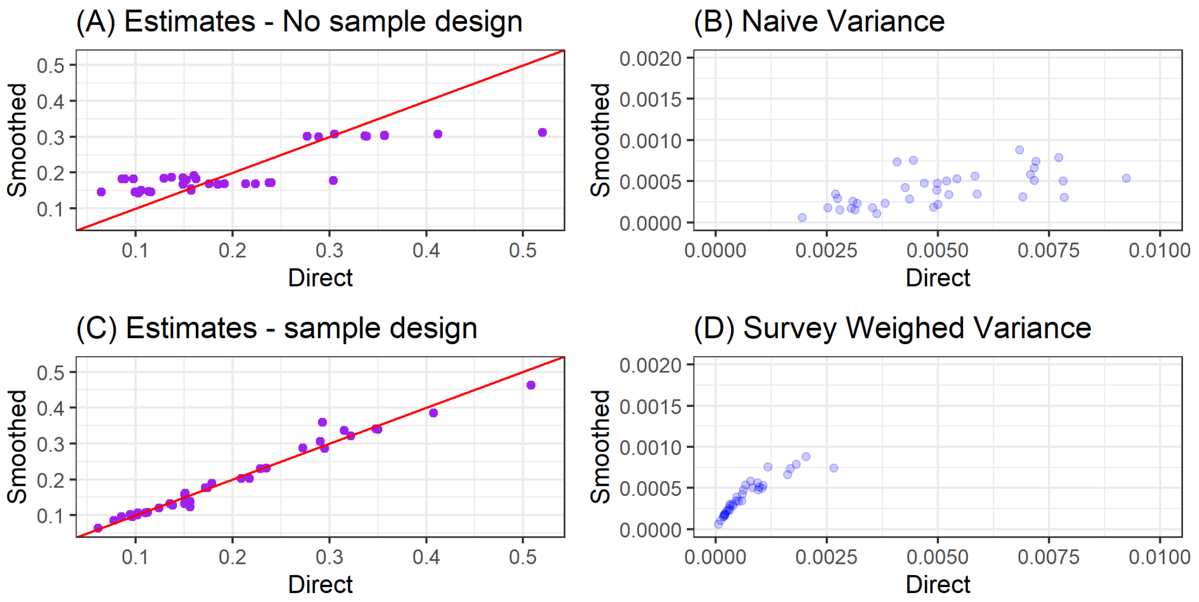


*Figure 6: Comparison of variance with estimate obtained using smoothing of the sample design. Panel (A) and Panel (B) show the estimation obtained without considering the design, and panel (C) and Panel (D) are the estimation obtained when the survey design included. For each plot the direct model is plotted against the smoothed model.*

The formula is provided in the supplementary document suggested to approximate the weight, including the correction for non-response between the clusters was as follows:

$$W_{hi} = \frac{A_h f_{hi}^{\alpha}}{a_h^c}$$

With the variation factor $\boldsymbol{f^{\alpha}} = \frac{\boldsymbol{d_{hi}^{HH}}}{\frac{\boldsymbol{A_h Mb_h}}{\boldsymbol{a_h^c s_h}}}$, $\boldsymbol{\alpha} \in [\mathbf{0}, \mathbf{1}]$ and $\boldsymbol{d_{hi}^{HH}} = \frac{\boldsymbol{M}}{\boldsymbol{m^c}}$ the denormalized type of the household weight provided in the DHS data as HV005 variable. $\boldsymbol{h}$ is the stratum from $\mathbf{1}$ to $\mathbf{20}$, which represents the number of strata by rural and urban area for all the regions used as stratification, and $\boldsymbol{i}$ represents the cluster. $\boldsymbol{A_h}$ is the total number of clusters from the census in the stratum $\boldsymbol{h}$, $\boldsymbol{a_h^c}$ is the number of clusters interviewed in stratum $\boldsymbol{h}$, $\boldsymbol{Mb_h}$ the average number of households by sampling strata for each cluster from the census data found in Table A.3 of the Gabon DHS final report, $\boldsymbol{m^c}$ the number of households completed, and $\boldsymbol{M}$ is the number of total households according to the census frame. For this work, $\boldsymbol{\alpha}$ was set to 0.5. The sensitivity analysis could be performed for the other values **[29]**.

***Table 5**: Space-time smoothing estimation of malaria prevalence*

| Region | Prevalence 2000 - 95% CI | Prevalence 2005 - 95% CI | Prevalence 2010 - 95% CI | Prevalence 2015 - 95% CI |
|---|---|---|---|---|
| Estuaire | 0.46 (0.42 - 0.51) | 0.16 (0.12 - 0.20) | 0.15 (0.12 - 0.18) | 0.29 (0.25 - 0.32) |
| Moyen-Ogooue | 0.38 (0.34 - 0.43) | 0.13 (0.10 - 0.16) | 0.11 (0.08 - 0.13) | 0.23 (0.19 - 0.27) |
| Woleu-Ntem | 0.36 (0.30 - 0.42) | 0.13 (0.08 - 0.18) | 0.13 (0.09 - 0.17) | 0.23 (0.18 - 0.28) |
| Ngounie | 0.34 (0.30 - 0.38) | 0.10 (0.07 - 0.12) | 0.11 (0.08 - 0.13) | 0.20 (0.17 - 0.24) |
| Ogooue-Ivindo | 0.34 (0.29 - 0.39) | 0.10 (0.07 - 0.13) | 0.10 (0.07 - 0.12) | 0.20 (0.16 - 0.25) |
| Ogooue-Maritime | 0.34 (0.28 - 0.39) | 0.12 (0.10 - 0.14) | 0.06 (0.05 - 0.08) | 0.19 (0.15 - 0.23) |
| Ogooue-Lolo | 0.32 (0.27 - 0.38) | 0.09 (0.05 - 0.12) | 0.10 (0.07 - 0.13) | 0.18 (0.13 - 0.22) |
| Haut-Ogooue | 0.31 (0.26 - 0.35) | 0.12 (0.07 - 0.18) | 0.10 (0.07 - 0.13) | 0.16 (0.13 - 0.19) |
| Nyanga | 0.29 (0.25 - 0.32) | 0.14 (0.10 - 0.18) | 0.11 (0.08 - 0.13) | 0.18 (0.15 - 0.20) |

***Table 6**: Space-time smoothing estimation prevalence by year, province and type of residence from the unit level model*

| Province | Type of residence | Prevalence 2000 - 95% CI | Prevalence 2005 - 95% CI | Prevalence 2010 - 95% CI | Prevalence 2015 - 95% CI |
|---|---|---|---|---|---|
| Estuaire | U | 0.45 (0.43 - 0.47) | 0.12 (0.10 - 0.14) | 0.13 (0.12 - 0.14) | 0.26 (0.25 - 0.27) |
| | R | 0.55 (0.54 - 0.57) | 0.18 (0.16 - 0.20) | 0.17 (0.16 - 0.18) | 0.33 (0.32 - 0.34) |
| Haut-Ogooue | U | 0.26 (0.24 - 0.28) | 0.13 (0.11 - 0.15) | 0.09 (0.08 - 0.10) | 0.13 (0.12 - 0.14) |
| | R | 0.36 (0.34 - 0.38) | 0.19 (0.17 - 0.21) | 0.13 (0.12 - 0.14) | 0.20 (0.19 - 0.21) |
| Moyen-Ogooue | U | 0.35 (0.34 - 0.37) | 0.10 (0.08 - 0.12) | 0.08 (0.07 - 0.09) | 0.20 (0.19 - 0.21) |
| | R | 0.45 (0.44 - 0.47) | 0.16 (0.15 - 0.18) | 0.12 (0.11 - 0.13) | 0.27 (0.26 - 0.28) |

| Province | Type of residence | Prevalence 2000 - 95% CI | Prevalence 2005 - 95% CI | Prevalence 2010 - 95% CI | Prevalence 2015 - 95% CI |
|---|---|---|---|---|---|
| Ngounie | U | 0.30 (0.29 - 0.32) | 0.06 (0.04 - 0.08) | 0.09 (0.08 - 0.10) | 0.18 (0.16 - 0.19) |
| | R | 0.40 (0.39 - 0.42) | 0.12 (0.10 - 0.14) | 0.13 (0.12 - 0.14) | 0.25 (0.24 - 0.26) |
| Nyanga | U | 0.23 (0.21 - 0.25) | 0.13 (0.11 - 0.15) | 0.09 (0.09 - 0.10) | 0.14 (0.13 - 0.15) |
| | R | 0.33 (0.31 - 0.35) | 0.19 (0.17 - 0.21) | 0.14 (0.13 - 0.15) | 0.21 (0.20 - 0.23) |
| Ogooue-Ivindo | U | 0.30 (0.28 - 0.32) | 0.06 (0.04 - 0.08) | 0.07 (0.07 - 0.08) | 0.18 (0.17 - 0.19) |
| | R | 0.40 (0.38 - 0.42) | 0.13 (0.11 - 0.14) | 0.12 (0.11 - 0.13) | 0.25 (0.24 - 0.26) |
| Ogooue-Lolo | U | 0.28 (0.26 - 0.30) | 0.05 (0.03 - 0.07) | 0.08 (0.07 - 0.09) | 0.14 (0.13 - 0.16) |
| | R | 0.38 (0.36 - 0.40) | 0.11 (0.10 - 0.13) | 0.12 (0.11 - 0.13) | 0.21 (0.20 - 0.23) |
| Ogooue-Maritime | U | 0.28 (0.26 - 0.29) | 0.09 (0.07 - 0.11) | 0.04 (0.03 - 0.05) | 0.15 (0.14 - 0.16) |
| | R | 0.38 (0.36 - 0.39) | 0.15 (0.14 - 0.17) | 0.08 (0.07 - 0.09) | 0.22 (0.21 - 0.23) |
| Woleu-Ntem | U | 0.25 (0.23 - 0.27) | 0.11 (0.09 - 0.13) | 0.13 (0.12 - 0.14) | 0.20 (0.18 - 0.21) |
| | R | 0.35 (0.33 - 0.37) | 0.17 (0.16 - 0.19) | 0.17 (0.16 - 0.18) | 0.27 (0.26 - 0.28) |

## 1.2 Linear mixed model and variable selection

### 1.2.1 Pairwise correlation

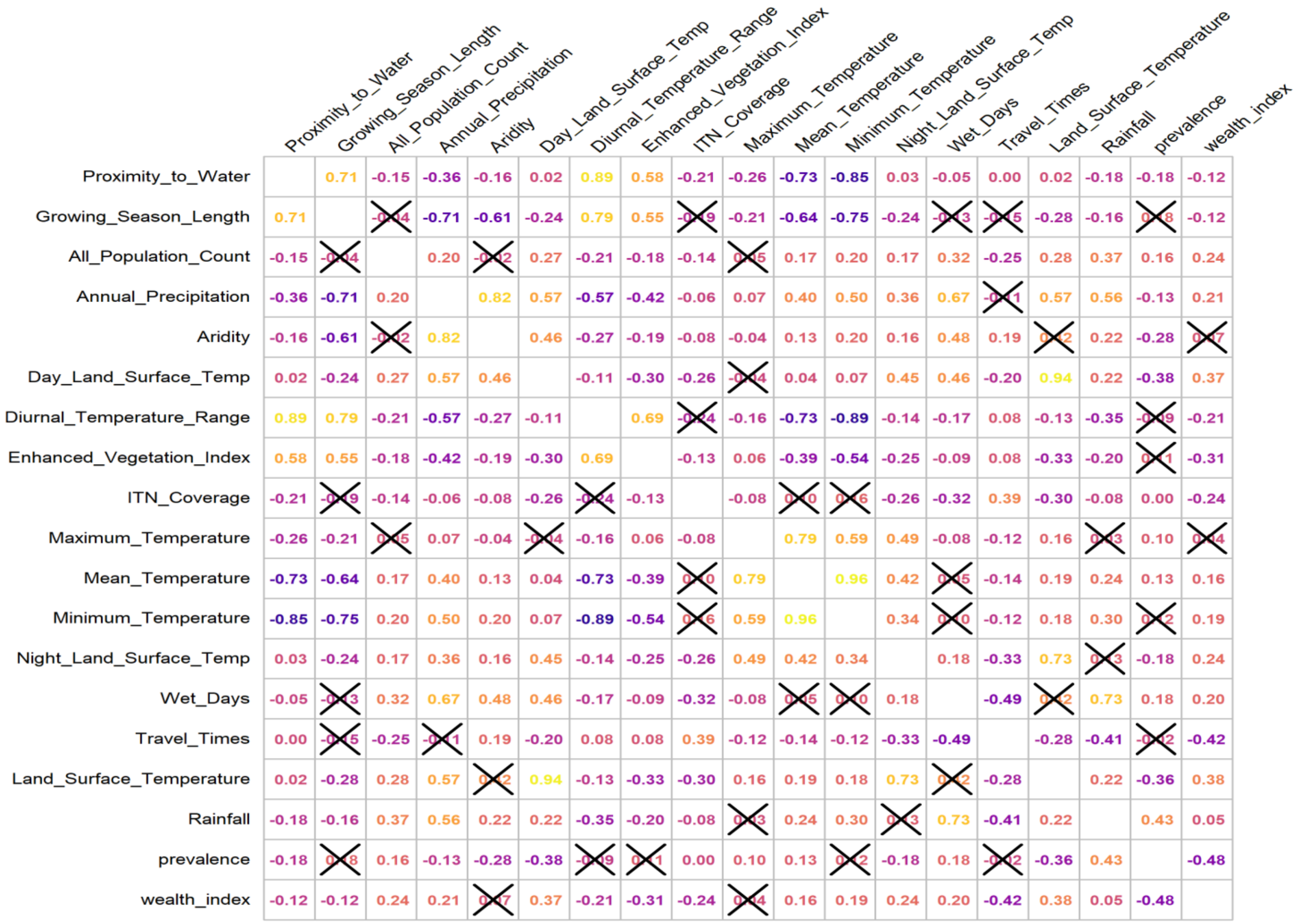


***Figure 7:*** *Correlation matrix estimating the correlation between the covariates and malaria prevalence. The black cross means no evidence of association between two variables*.

### 1.2.2 Model building

As shown in Table 7, the results suggested that using the model with the random slope on time improved the fit of the model significantly. The results need to be interpreted with caution, due to collinearity of the parameters and the assumptions.

***Table 7**: Random intercept vs random slope year*

| Term | Npar | AIC | statistic | df | p.value |
|---|---|---|---|---|---|
| Random intercept | 16 | -2,654.082 | | | |
| Random intercept + slope ITN | 18 | -2,846.571 | 196.4885 | 2 | < 0.001 |

### (1) Multi-collinearity

As shown in Table 8, collinearity was detected for the variables annual precipitation and aridity. The VIF was above 10, therefore annual precipitation was removed from the model. Also, the problem of multicollinearity was detected in the association of precipitation variable with diurnal day land temperature, land surface temperature, wet days and proximity to water. Therefore, precipitation was dropped from the model. The growing season was removed as there was some evidence of correlation with aridity as shown in the plot. However, aridity was used in the model because some studies have suggested an association with the prevalence of malaria (14). After the exclusion, the VIF was re-calculated for the remaining variables. Thus, in Table 8, the VIF was less than 10 for all the variables after removing some variables.

***Table 8**: Multicollinearity after removing variables*

| Covariates | VIF | VIF (After removing variables) |
|---|---|---|
| Proximity to Water | 7.44 | 6.87 |
| Growing Season Length | 5.44 | - |
| All Population Count | 1.65 | 1.72 |
| Annual Precipitation | 50.68 | - |
| Aridity | 27.10 | 7.23 |
| Day Land Surface Temp | 7.60 | 7.79 |
| Diurnal Temperature Range | 11.96 | 8.17 |
| Enhanced Vegetation Index | 2.47 | 2.80 |
| ITN Coverage | 5.53 | 4.15 |
| Mean Temperature | 3.63 | 3.33 |
| Wet Days | 7.43 | 6.40 |
| Land Surface Temperature | 8.16 | 8.71 |
| wealth index | 1.54 | 1.60 |
| Rainfall | 6.28 | 4.49 |

VIF = Variance Inflation Factor

### (2) Comparison of the models: day land surface and land surface temperature

After evaluating the multicollinearity, the following models were compared: **1)** model with covariates + day land temperature and **2)** model with covariates + land surface temperature. The idea was to reduce as much as possible the multicollinearity by removing potential variables which were not improving the fit of the model. As a result, from the Table 9, the model with day land surface temperature was the best as the AIC was the lowest.

***Table 9****: Model + Day land surface vs Model + Land surface temperature - AIC*

| Model | AIC |
|---|---|
| Covariates + Day Land surface temperature | -2836.79 |
| Covariates + Land surface temperature | -2825.85 |

**(3) comparing the models: including and excluding diurnal temperature**

In Table 10, the AIC for the model without diurnal temperature was the lowest. Hence, diurnal temperature was excluded from the model. The following variables were the final variables selected: *Proximity to Water, all population count, aridity, day land surface temperature, enhanced vegetation index, ITN coverage, mean temperature, wealth index, wet days and rainfall*. For this analysis the main variables were ITNs coverage, rainfall, aridity and wet days.

***Table 10**: model with diurnall vs without diurnal*

| Model | AIC |
|---|---|
| Covariates + Diurnal temp | -2836.79 |
| Covariates - Diurnal temp | -2838.78 |

**(4) Varying the intercept and slope on ITN coverage and year**

After selecting this model, to assess if the fit of the model was better when the random slope is introduced on the variable year and ITNs coverage, the two models were compared. In Table 11, the model including slopes on both ITNs coverage and year was better than the model including the random slope only on year (ANOVA; p-value < 0.05). The random slope was considered only for the variables ITN and year. In fact, if rainfall, aridity and the other variables were used with a random slope, the number of observations in the data would not allow to fit a good model. Also, according to our description, ITNs coverage appeared to be the most varying in space and time.

***Table 11**: Random slope year vs Random slopes ITN + year*

| Term | npar | AIC | Statistic | df | P-value |
|---|---|---|---|---|---|
| Random slope ITN | 15 | -2838.78 | NA | | |
| Random slopes ITN + year | 17 | -2867.35 | 32.57 | 2 | < 0.001 |

### 1.2.3 Diagnostics of the selected model

As shown in Figure 8, the variance was not constant as the vertical spread was not the same for different values on the x-axis. The probability normal plot either for the random effect or the residuals showed that there was a slight departure from normal distribution as points were more deviating from the ideal line. However, the log transformation was better in terms of reducing the variance to become more constant, and stretching the points better than the untransformed data. The departure from the normal distribution either for the random effect or the residuals was reduced.

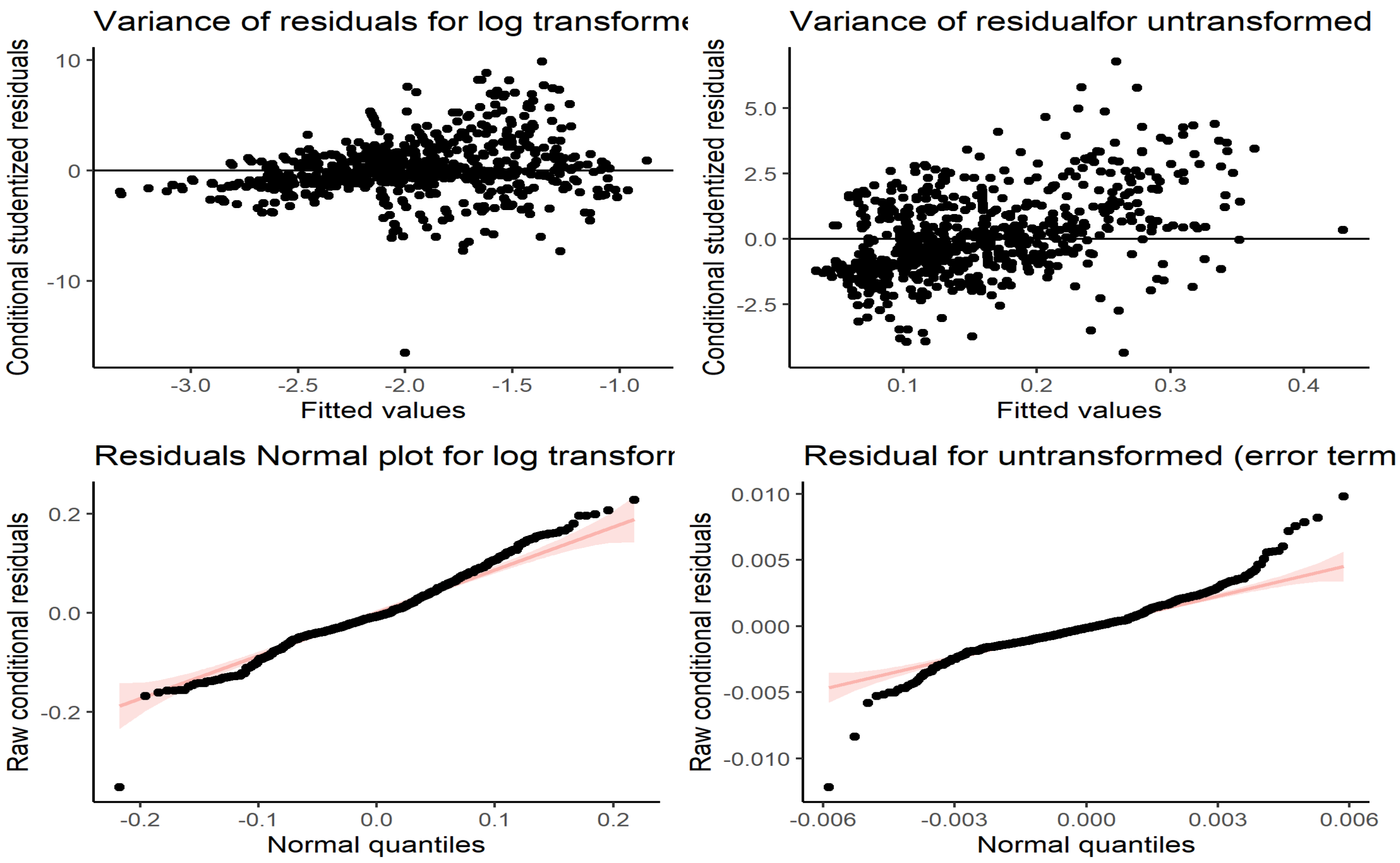


***Figure 8**: Comparison of residuals from the log transformed and the untransformed prevalence. The plot at the left and right represents the residual variance of log transformed and for untransformed malaria prevalence respectively*

As shown in Figure 9, after using the square root transformation, the residuals and the random effects became more normal, and the variance became more constant than observed with the log transformation. Using the inverse square root transformation, the random effect for year and ITN did not approximate the normal distribution more than the square root transformation (see appendix table (**Erreur ! Source du renvoi introuvable.**).

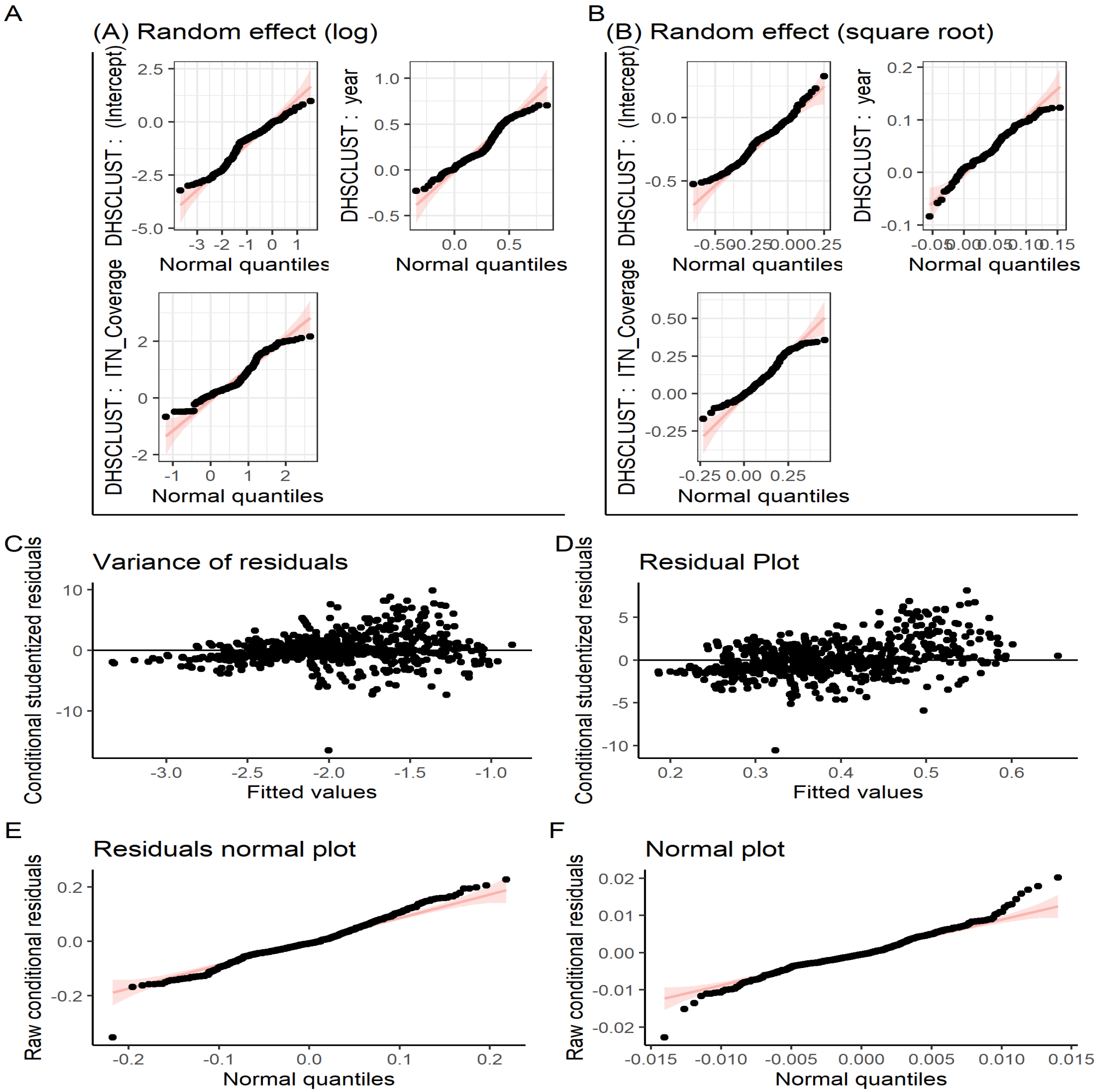


***Figure 9****: Comparison of residual's variance and distribution for the diagnostic of the model between log and square root transformation. Panel A and B represent the random effects, C to E the residuals of model using the log, D and F the residual of the model using square root transformation on the outcome.*

**Final Conclusion:**

Despite little deviation from the assumption, the final model was interpreted using either the log or the square root transformation, because the linear model is robust for small deviations. The interest here was in the random effect, hence the model with square root transformation could be used. Indeed, with this model, the assumptions were less violated for the random effect (68). According to this model (Figure 10), the average effect of the prevalence was different from cluster to cluster when not adjusting for the other variables. This result was significant because the credible interval for the intercept did not contan 0. The effect of year and ITNs coverage on the prevalence by cluster was significant. It was represented by those black points who did not cross the red line with their credible interval. Using the model with log transformation, the same result was also observed. However, no significant effect on the year was obtained for the model using the inverse square root transformation (see appendix).

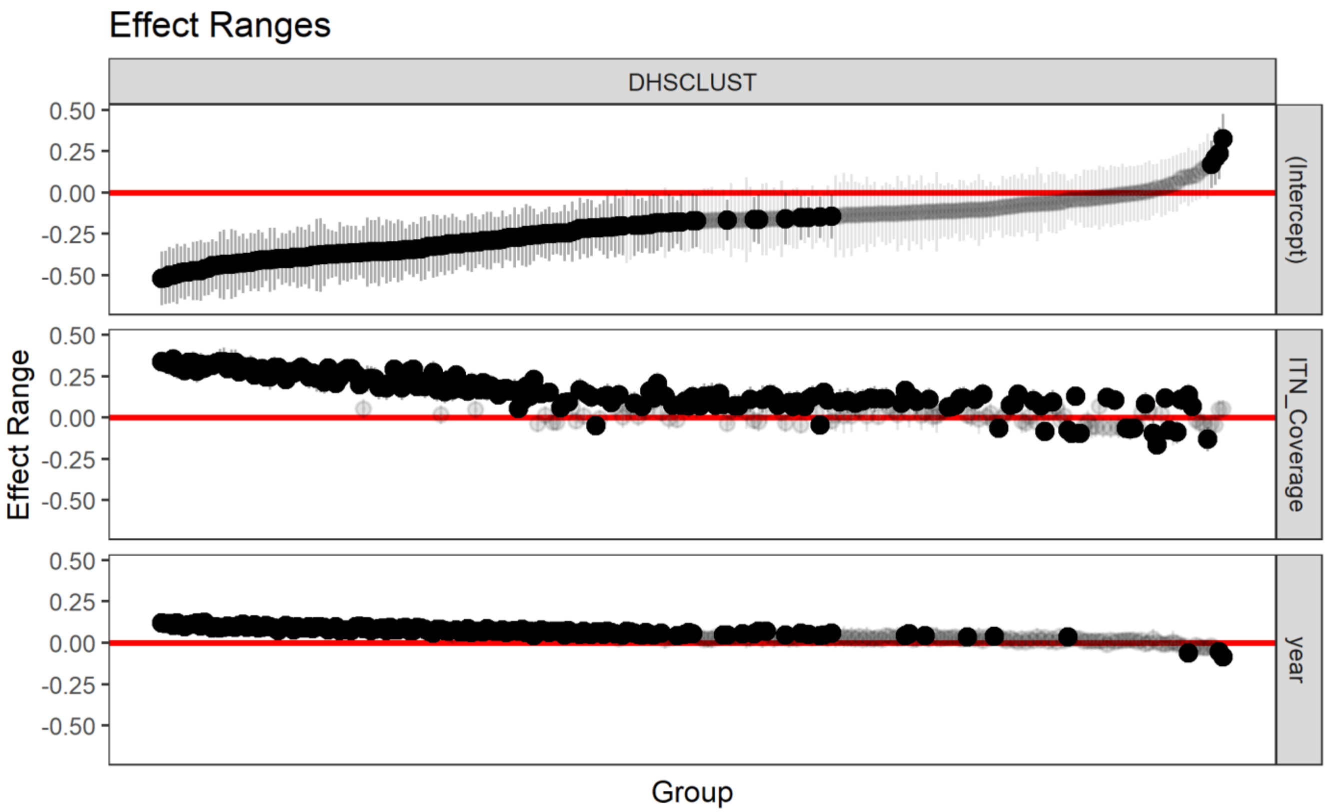


***Figure 10**: Effect of ITNs coverage and year on the prevalence from each cluster*

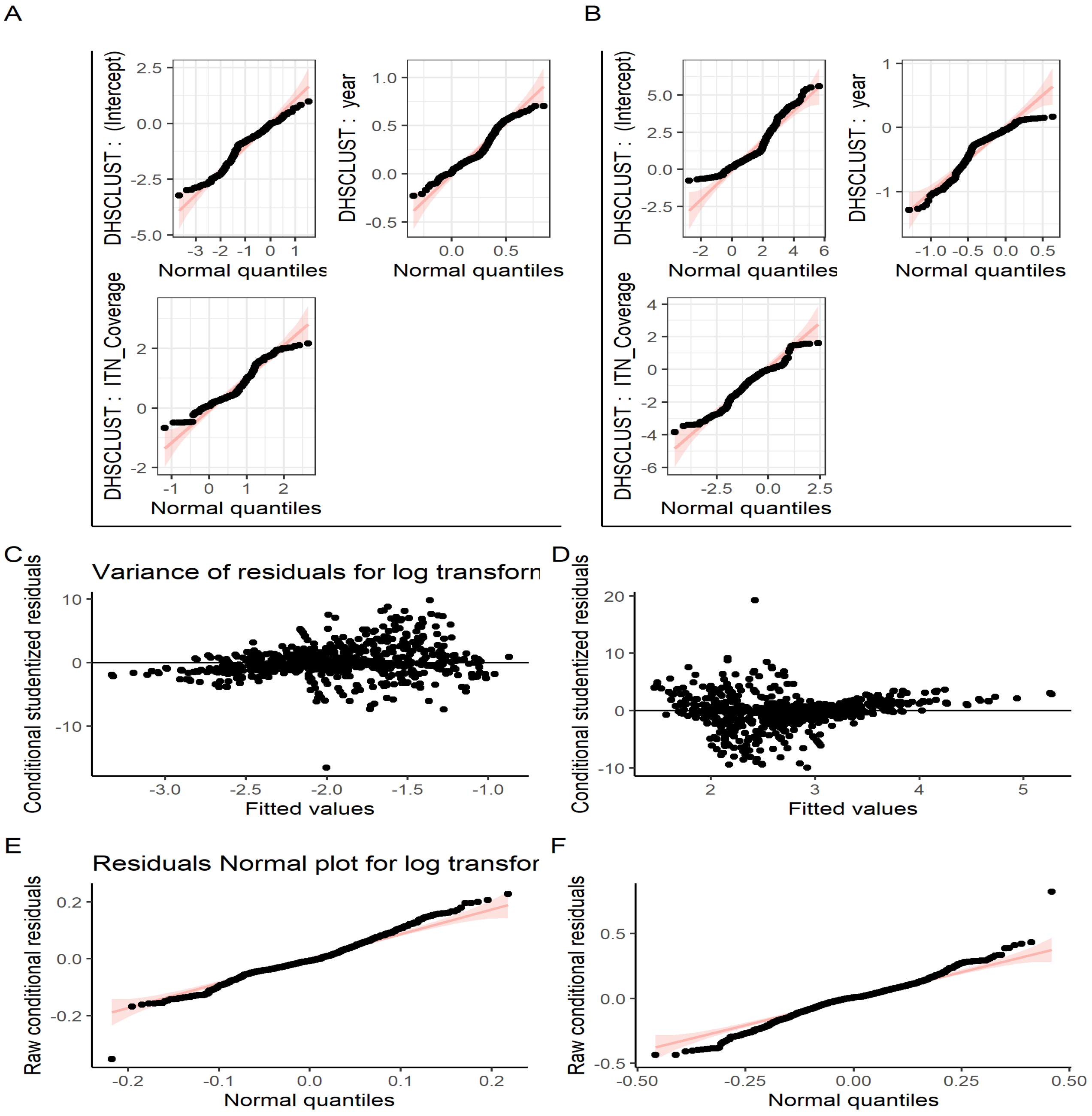


**Figure 11**: Log transformation vs 1/square root

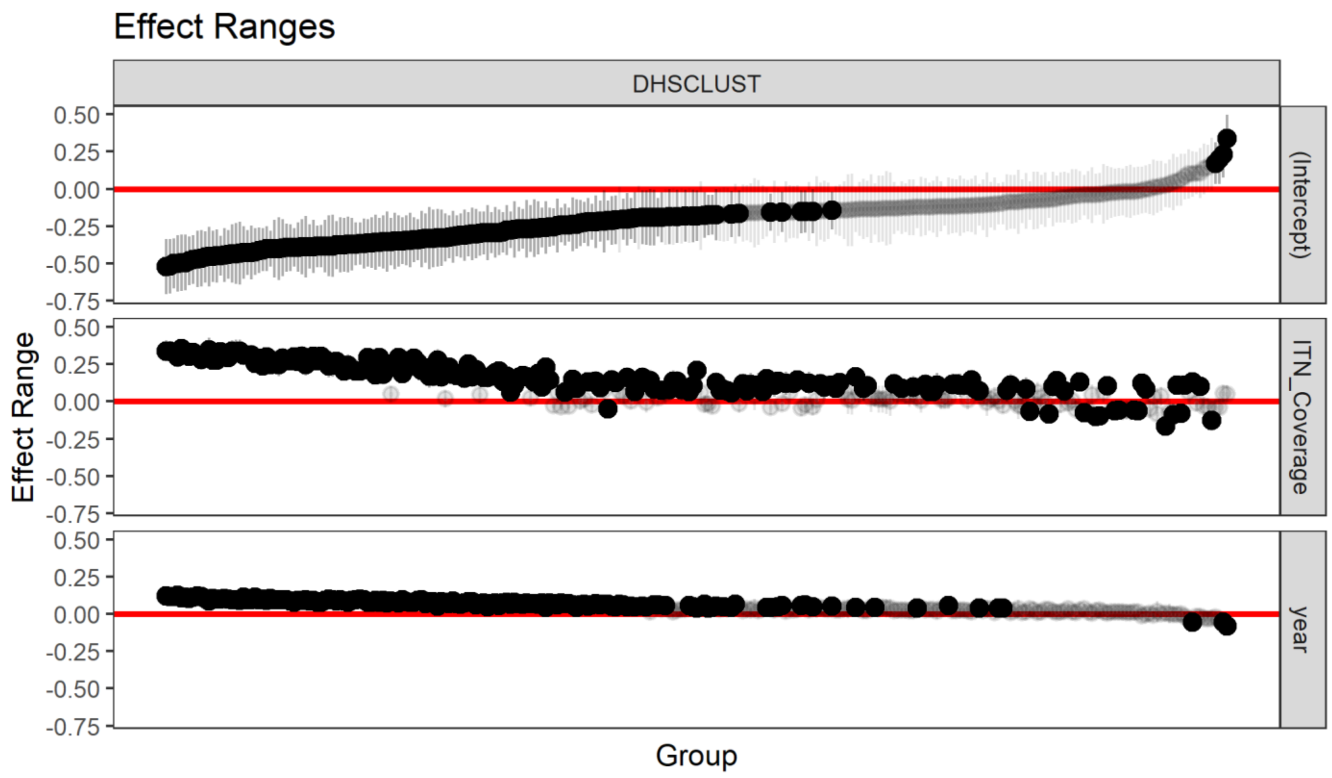


***Figure 12:*** *Effect of ITN and year on the prevalence from each cluster: square root transformation of malaria prevalence*

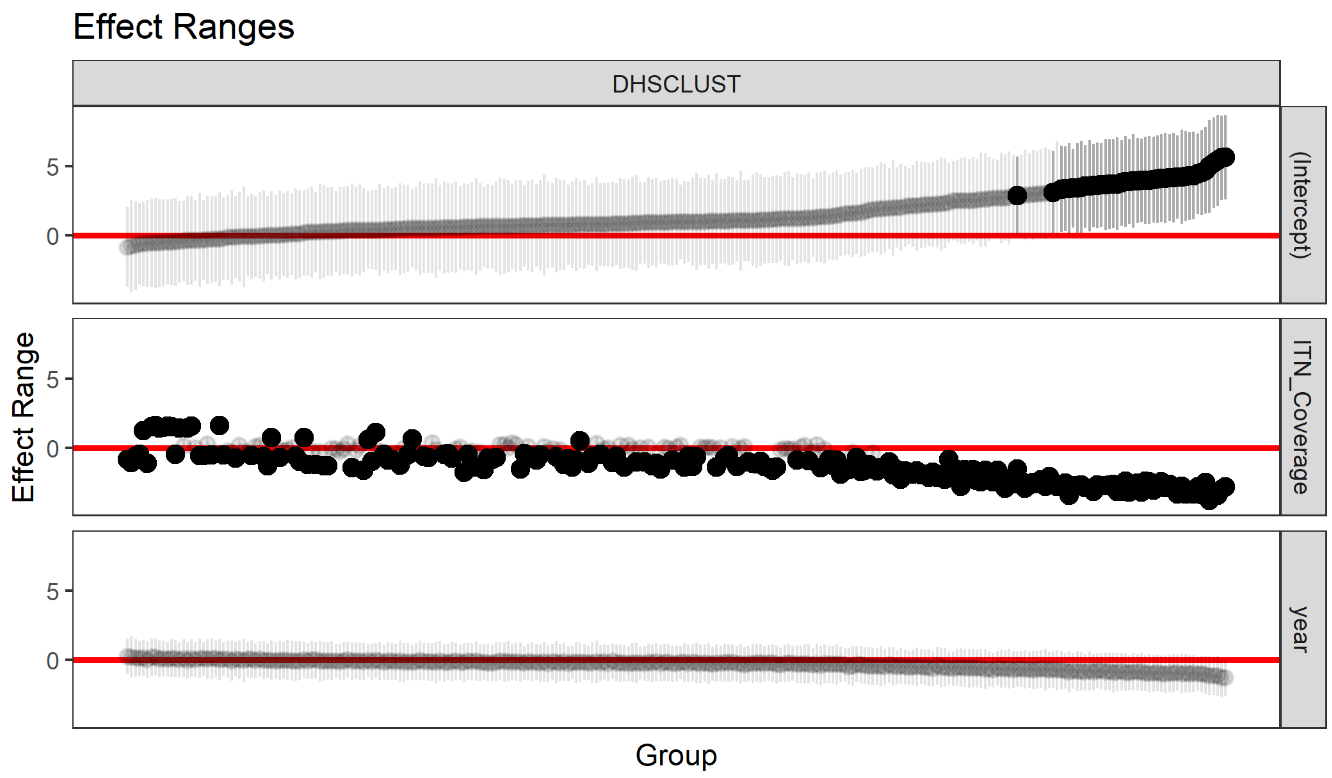


***Figure 13**: Effect of ITN and year on the prevalence from each cluster: inverse square root transformation of malaria prevalence*

## 1.3 Multiple linear regression of malaria prevalence

Multiple linear regression for different years was performed using variables selected previously. However, as there was no data available for the variables ITNs coverage and all population count for the year 2000, the linear regression was conducted for this year without these two variables. The results with and without extreme values found on the variable population count were compared. The results of the regression were not modified except the coefficient which became significant after removing the extreme values. Hence, all the analysis was performed without these values. For all the years, the F-test showed with overwhelming evidence that at least one variable was associated with the outcome among all the variables selected. The assumption of linearity was assumed looking to the scatterplot presented in **Erreur ! Source du renvoi introuvable.**.

After using either the log or square root transformation, the assumption of normality and homoscedasticity were met (Figure 14).

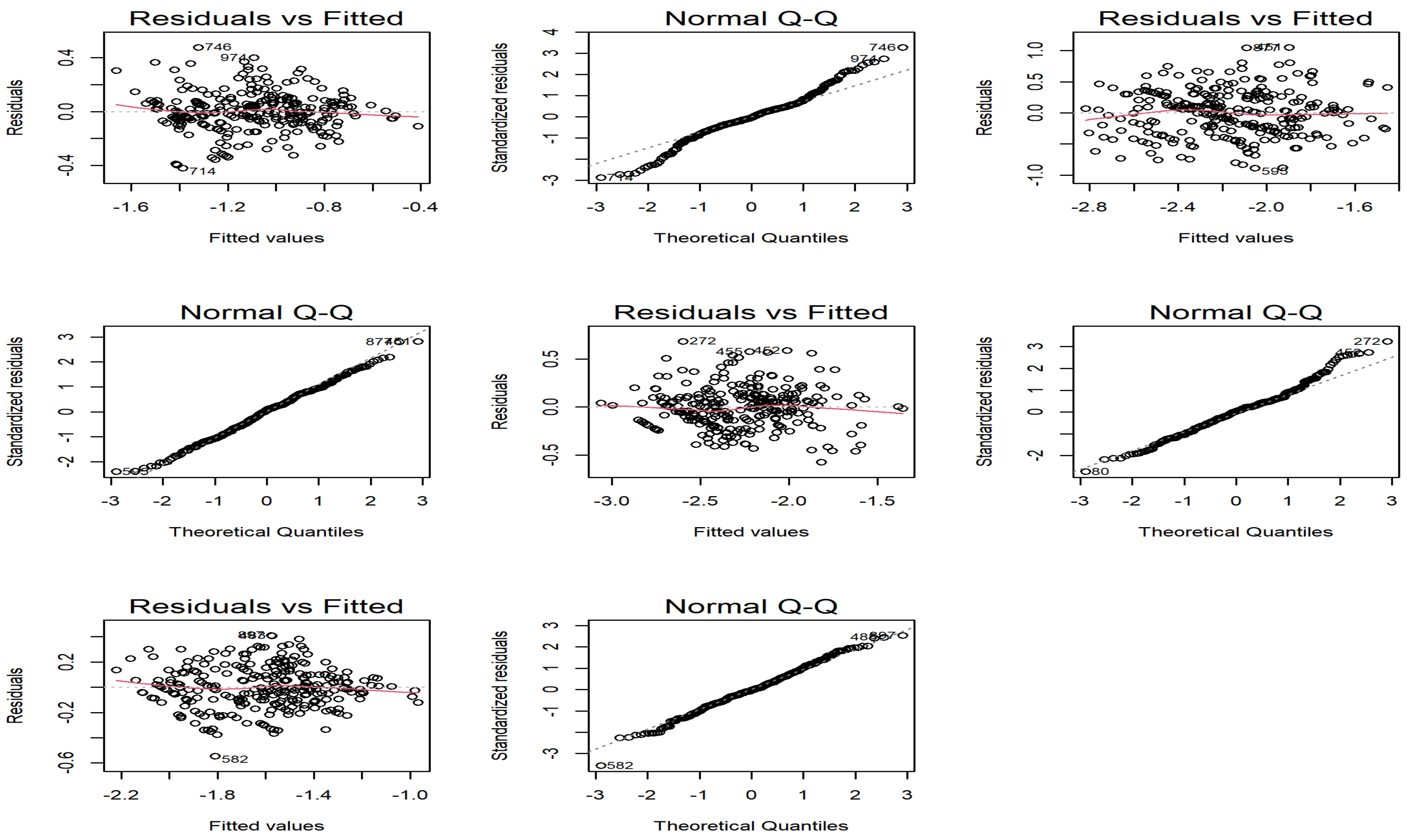


***Figure 14**: Residuals inspection: The pair of plots represent the diagnostic for each year respectively 2000, 2005, 2010 and 2015*

For the assumption of independence on the residuals, overwhelming evidence of strong spatial autocorrelation in the residuals for each year was found in Table 12: Spatial autocorrelation on residuals and malaria prevalence over time. After including variables to

explain the variation of the prevalence, the spatial autocorrelation observed on the prevalence was slightly reduced but not enough. A small effect of time was observed from the year 2000 to 2005, 2005 to 2010, 2010 to 2015, that is, a difference of spatial autocorrelation of 0.12, 0.26, and 0.06 respectively. This difference was almost constant when considering only the dependent variable.

***Table 12**: Spatial autocorrelation on residuals and malaria prevalence over time*

| year | Moran Index – outcome | statistic 1 | P-value | Moran Index - residuals | statistic 2 | P-value |
|---|---|---|---|---|---|---|
| 2000 | 0.88 | 11.81 | <0.001 | 0.71 | 9.66 | <0.001 |
| 2005 | 0.87 | 11.62 | <0.001 | 0.84 | 11.18 | <0.001 |
| 2010 | 0.83 | 11.04 | <0.001 | 0.57 | 7.71 | <0.001 |
| 2015 | 0.83 | 11.12 | <0.001 | 0.62 | 8.31 | <0.001 |

This OLS regression showed that ITNs coverage was significantly associated with the prevalence, in such a way that one-unit increase was associated with 10% decrease of the prevalence in a linear fashion. Its magnitude was varying slightly by year. The prevalence was increasing by 2% with a one unit increased of wet days. Wealth index was also increasing with the prevalence. In fact, almost all the variables were significantly related with the prevalence of malaria. It was also noted that the coefficients were varying slightly by year (Table 13).

*Table 13: Ordinary least square regression for each year*

| Variables | Estimate 1 - 2000 | | Estimate 2 – 2005 | | Estimate 3 - 2010 | | Estimate 4 - 2015 | |
|---|---|---|---|---|---|---|---|---|
| | Beta | P-value | Beta | P-value | Beta | P-value | Beta | P-value |
| (Intercept) | -4.9 | < 0.001 | -0.70 | 0.64 | -0.92 | 0.31 | -0.04 | 0.95 |
| ITN coverage | | | -1.74 | < 0.001 | -1.87 | < 0.001 | -1.02 | < 0.001 |
| Mean temperature | 0.13 | < 0.001 | -0.09 | 0.10 | -0.06 | 0.06 | -0.01 | 0.71 |
| Wet days | 0.12 | < 0.001 | 0.28 | < 0.001 | 0.26 | < 0.001 | 0.08 | < 0.001 |
| Night land surface temp | | | 0.06 | 0.17 | 0.03 | 0.19 | 0.00 | 0.90 |
| Day land surface temp | -0.044 | < 0.001 | -0.01 | 0.52 | -0.05 | < 0.001 | -0.07 | < 0.001 |
| Wealth index | -0.0000007 | 0.0018 | 0.00 | < 0.001 | 0.00 | < 0.001 | 0.00 | < 0.001 |
| Rainfall | 0.019 | < 0.001 | 0.02 | 0.16 | 0.01 | 0.31 | 0.05 | < 0.001 |
| Proximity to water | 0.0078 | < 0.001 | -0.02 | < 0.001 | -0.01 | < 0.001 | -0.01 | < 0.001 |
| Enhanced vegetation index | -0.0011 | 0.56 | 0.01 | 0.31 | 0.01 | < 0.001 | 0.00 | 0.57 |
| All population count | | | -0.10 | < 0.001 | -0.06 | < 0.001 | -0.04 | < 0.001 |
| Aridity | -0.0047 | 0.069 | -0.06 | < 0.001 | -0.03 | < 0.001 | -0.02 | < 0.001 |

## 1.4 Geographical Weighted Regression

*Table 14: Diagnostic of GWR method*

| Diagnostics for local multicollinearity | | | | Diagnostics on spatial autocorrelation | |
|---|---|---|---|---|---|
| model | Rsquared (Min - Max) | local CN (Min - Max) | VIF (Min - Max) | Moran's I index | P-value |
| All values | 0.8-0.97 | 295.67-297.89 | 1.53-5.17 | 0.10 | 0.08 |

## 1.5 Spatial varying coefficients

No evidence was found to support that the relationship for some variables was varying by location. However, only day land surface temperature and ITNs coverage were plotted to represent the variation showed in Figure 15. All the other variables were not showing any pattern (see supplementary). The patterns observed assumed that the increase of the ITNs coverage was associated with the

decrease of the prevalence in the center of the country, and a slight increase around. An increase of the day land surface temperature had was associated with the decrease of  the prevalence in the center part. But they were not significant.

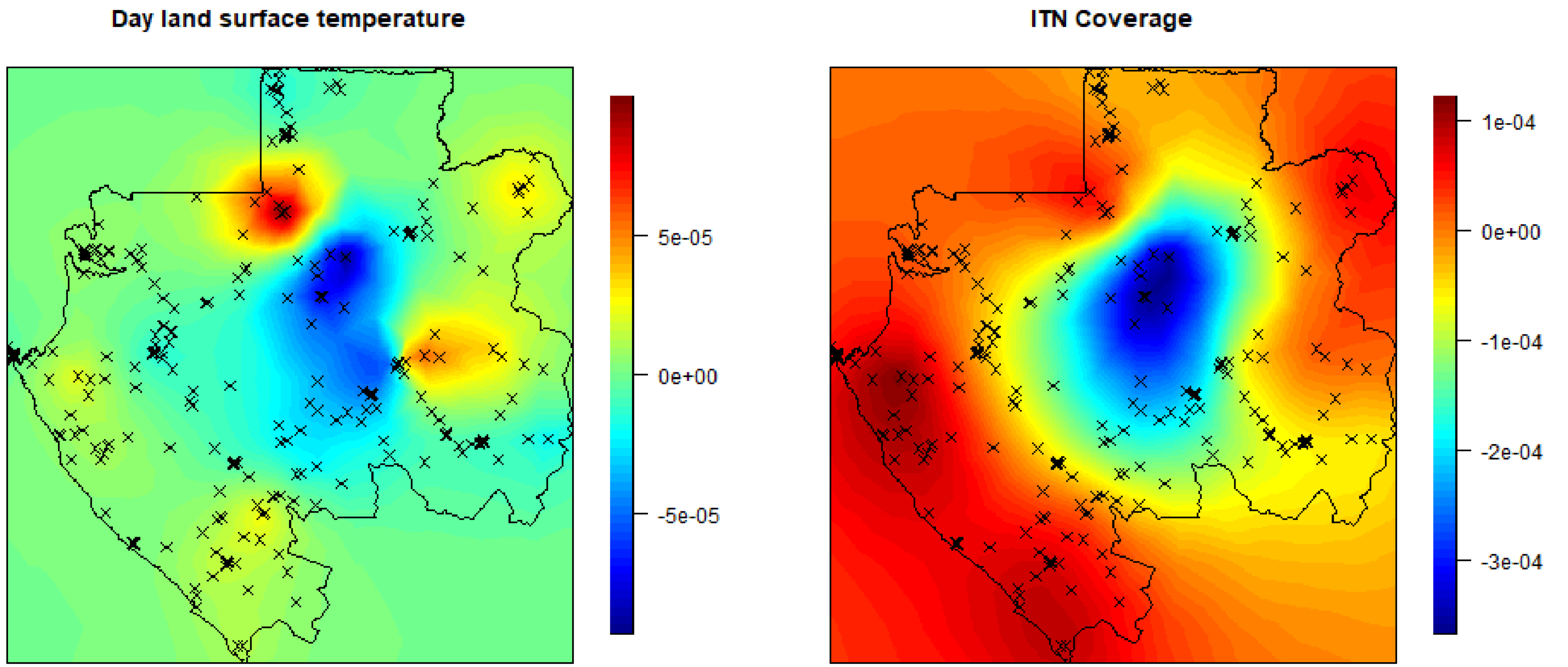


***Figure 15**: Spatial varying coefficient - visualization of the prevalence of malaria with Day land surface temperature (left) and ITN (right)*

### Spatio-temporal model

### Linear models

From M1, after adjusting with M2, M3 (enviromental variables), and M4 (full model), the spatial variation was reduced by 68%, 88%, and 76% (26%, 9%, and 27%) for SPTOLS (space-time correlated GAM) respectively (Table **15**). The best model with small DIC was the full model. The value of the range showed a strong spatial correlation decreasing slowly up to 142 km (290 km with GAM). For all the sub-models, the spatial variance was obviously greater than the measure error by more than 95%. The coefficients for time ($\rho$) were almost the same for all the three sub-models. No evidence for the time effect was found since the credible interval contains 0. The coefficient for time was too high and the credible interval too tight. For the replicated GAM, M3 was better than M2, while for the space-time correlated model, M2 was better than M3 with the lowest DIC. Using the full model, increasing the ITNs coverage (or population) by 1%, decreased significantly (and slightly) malaria prevalence by about 52% (3%); increasing the night land temperature by 1-degree increased malaria prevalence by 3% (supplementary material). Since no evidence of the effect of the time on the prevalence was found, the model without time by taking the mean value for all the years was run.

***Table 15**: Spatial parameters GAM with all variables considered as non-linear*

| | | **Spatio-temporal OLS model** | | **GAM model with non-linear variables** | |
|---|---|---|---|---|---|
| **Model** | **Parameter** | **Mean (SD)** | **95% CI** | **Mean (SD)** | **95% CI** |
| M1 | Meas error | 0.000064 (0.0000077) | (0.00005,0.00008) | 0.00053 (0.000064) | 0.00042;0.00067 |
| | Spatial variance | 0.17 (0.036) | (0.11,0.25) | 11 (2.2) | 7;16 |
| | Range | 333 (35) | (270,406) | 332 (31) | 276;396 |
| | Time coefficient (a) | 0.97 (0.0044) | (0.96,0.98) | 0.99 (0.0016) | 0.98;0.99 |
| M2 | Meas error | 0.000026 (0.0000045) | (0.000018,0.000036) | 0.00047 (0.000062) | 0.00036;0.0006 |
| | Spatial variance | 0.055 (0.01) | (0.038,0.077) | 8.1 (1.7) | 5.2;12 |
| | Range | 154 (15) | (128,185) | 278 (27) | 229;336 |
| | Time coefficient (a) | 0.97 (0.0044) | (0.96,0.98) | 0.99 (0.0015) | 0.99;0.99 |
| M3 | Meas error | 0.000071 (0.000012) | (0.00005,0.000095) | 0.0006 (0.000082) | 0.00045;0.00077 |

| | | | | | |
|---|---|---|---|---|---|
| | Spatial variance | 0.021 (0.0053) | (0.012,0.033) | 10 (2.3) | 6.4;15 |
| | Range | 153 (21) | (114,197) | 342 (36) | 279;422 |
| | Time coefficient (a) | 0.93 (0.014) | (0.89,0.95) | 0.99 (0.0018) | 0.98;0.99 |
| M4 | Meas error | 0.000023 (0.0000043) | (0.000016,0.000032) | 0.00053 (0.000073) | 0.0004;0.00068 |
| | Spatial variance | 0.044 (0.011) | (0.026,0.068) | 8 (1.9) | 4.9;12 |
| | Range | 142 (17) | (111,176) | 290 (33) | 230;360 |
| | Time coefficient (a) | 0.96 (0.0062) | (0.95,0.97) | 0.99 (0.0016) | 0.98;0.99 |

*GAM = Geo-additive Model; OLS = Ordinary Least Squared

## Spatial econometric Model

### Computing models

Using the Moran index, or the Lagrange multiplier (LM), no evidence of spatial dependence was observed. The DICs of the spatial lag models were smaller than the DIC of the GAM ran previously (-3125.58 vs -1744.26). Based on the ML (Maximum Likelihood), the best model was the SDEM. The fit with ML and INLA, gave approximately the same values (Table **16**).

### Impact

As shown in Table 16, since the indirect effect of ITNs coverage was positive and significant, therefore for a particular cluster, increasing the ITNs coverage (day land surface) in its nearby clusters (same cluster) was found to be significantly associated with the decrease of the prevalence in this cluster by 21% (2%). An increase of wet days (mean temperature) in a cluster (nearby clusters) was associated with an increase of the prevalence in the cluster by 3% (2%). There was a significant difference of the prevalence between the type of residence.

***Table 16**: Comparison INLA and Maximum likelihood for SLM and SDM - impacts for SDEM*

| Covariates | SDEM from ML | | SDEM from INLA – 95% CI | |
|---|---|---|---|---|
| | Direct impact | Indirect impact | Direct impact | Indirect impact |
| Mean temperature | -0.0089 | 0.019 | -0.009 (-0.025;0.0069) | 0.019 (0.0012;0.037) |
| Wet days | 0.032 | -0.0016 | 0.032 (0.012;0.052) | -0.0018 (-0.023;0.02) |
| ITN coverage | 0.14 | -0.21 | 0.14 (-0.067;0.34) | -0.21 (-0.4; -0.0055) |
| Urban - rural | -0.041 | -0.0083 | -0.041 (-0.054; -0.028) | -0.0082 (-0.022;0.0052) |

From M0, the spatial variation was reduced by 33% , 13%, 8% when considering the M4, the model with environmental variables (M3) and M2, respectively. Observing particularly the curve for the ITNs coverage, it appeared that malaria prevalence started to decrease only after 20% of the coverage (supplementary material).

**(1) Replicated model (rho = 0)**

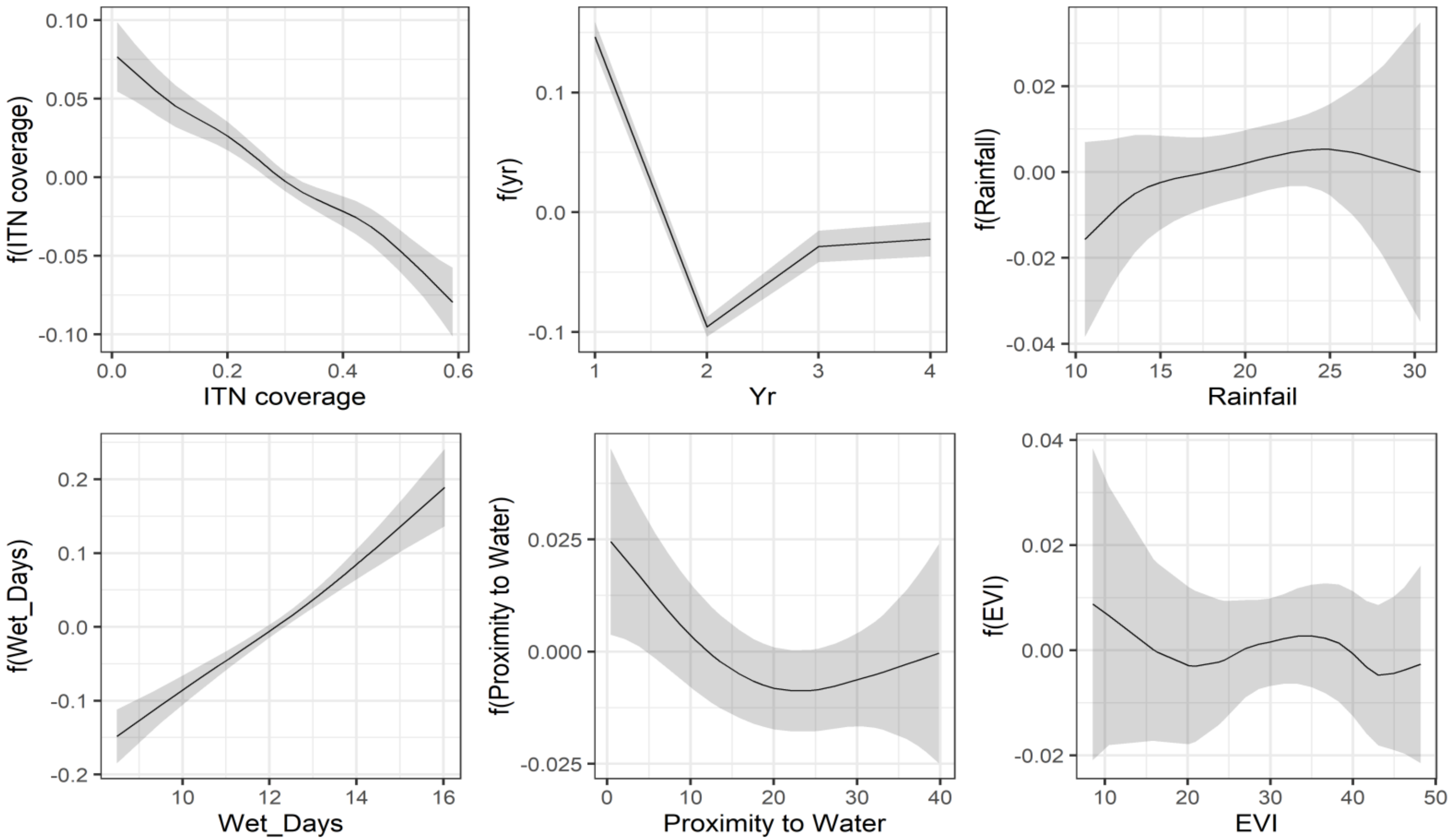


***Figure 16****: Smoother of malaria prevalence and covariates estimated from GAM with replicated method*

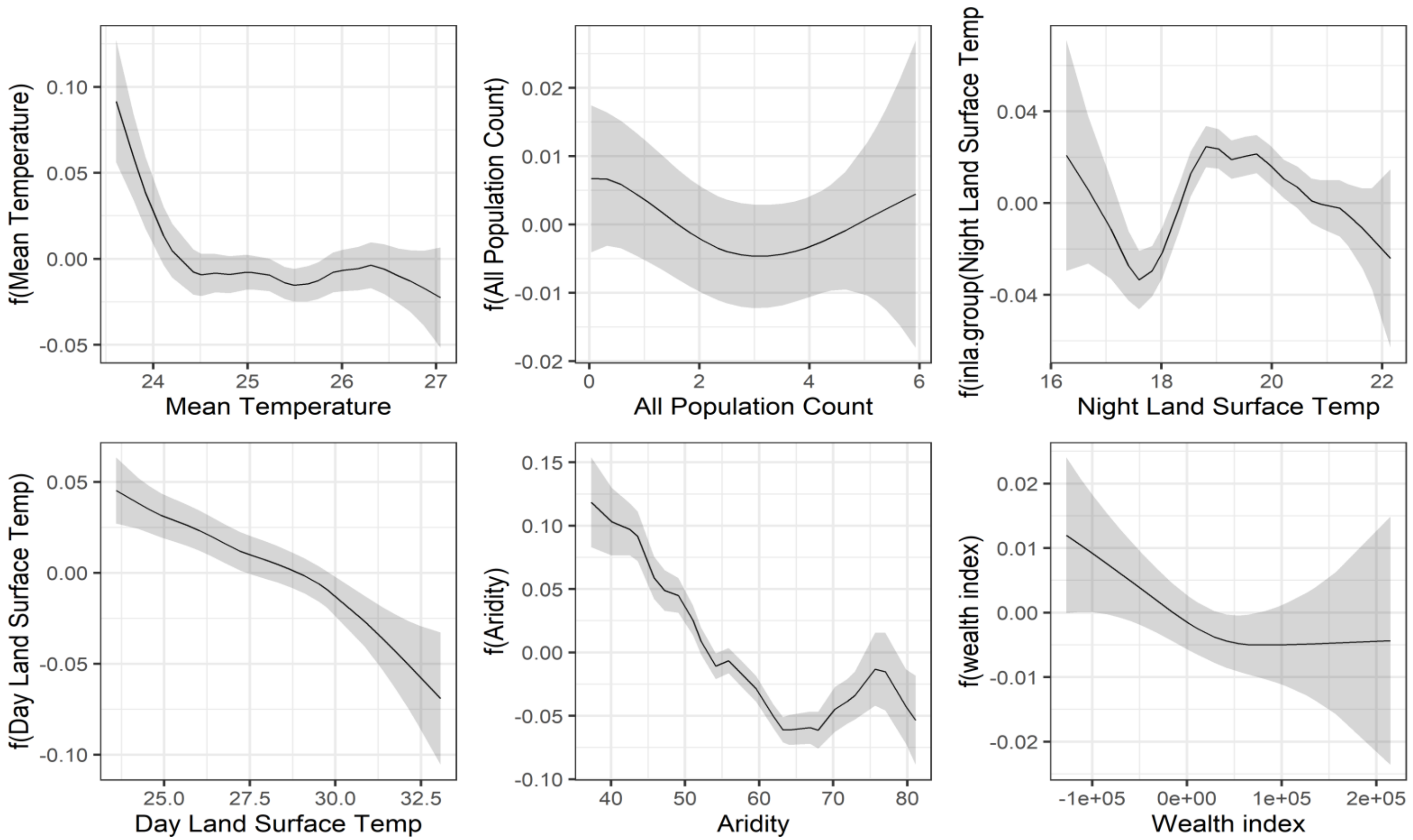


***Figure 17****: Smoother of malaria prevalence and covariates estimated from GAM with replicated method (cont)*

- **GAM on summarized data over year**

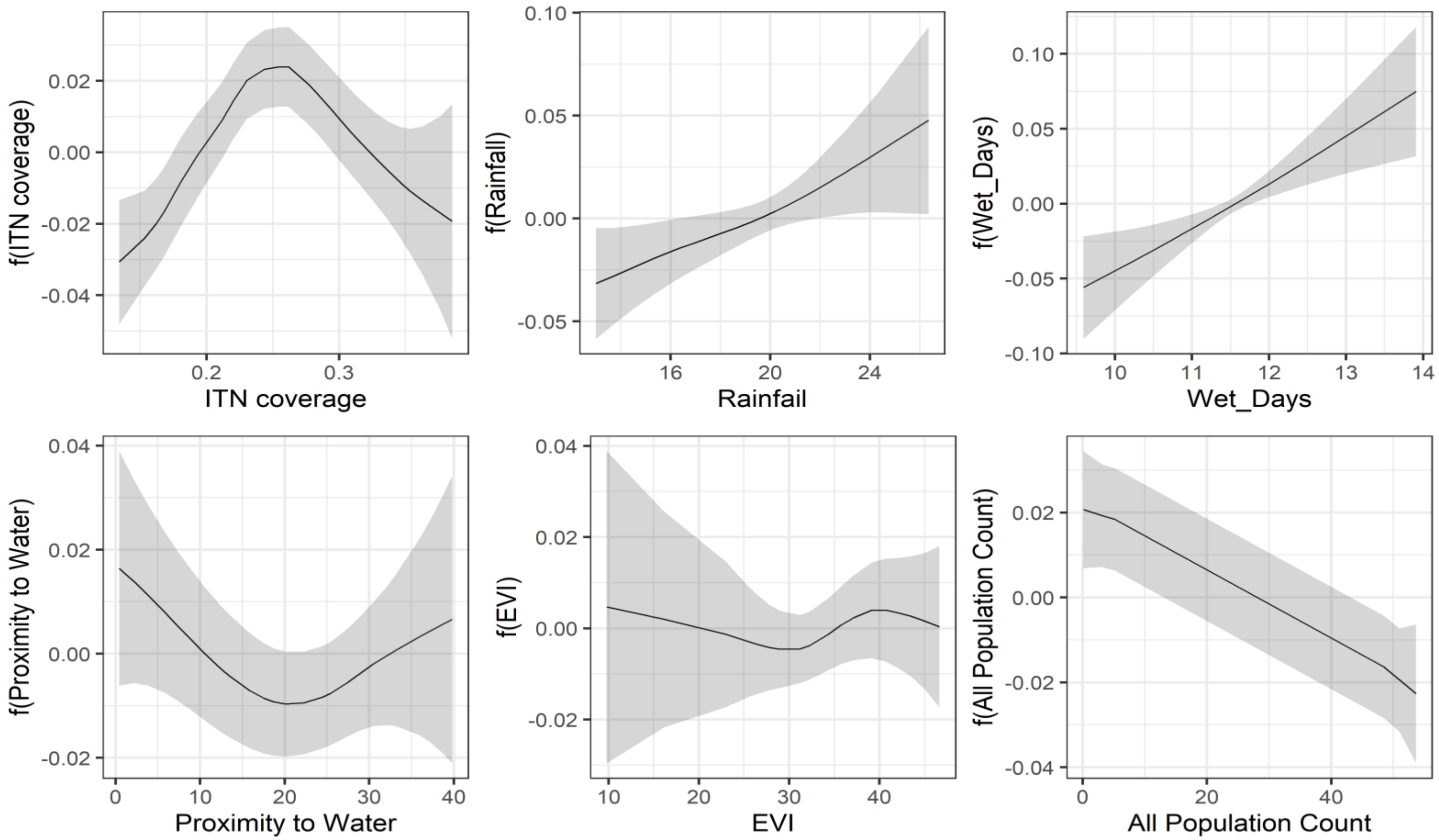


***Figure 18**: GAM on summarized data without year effect*

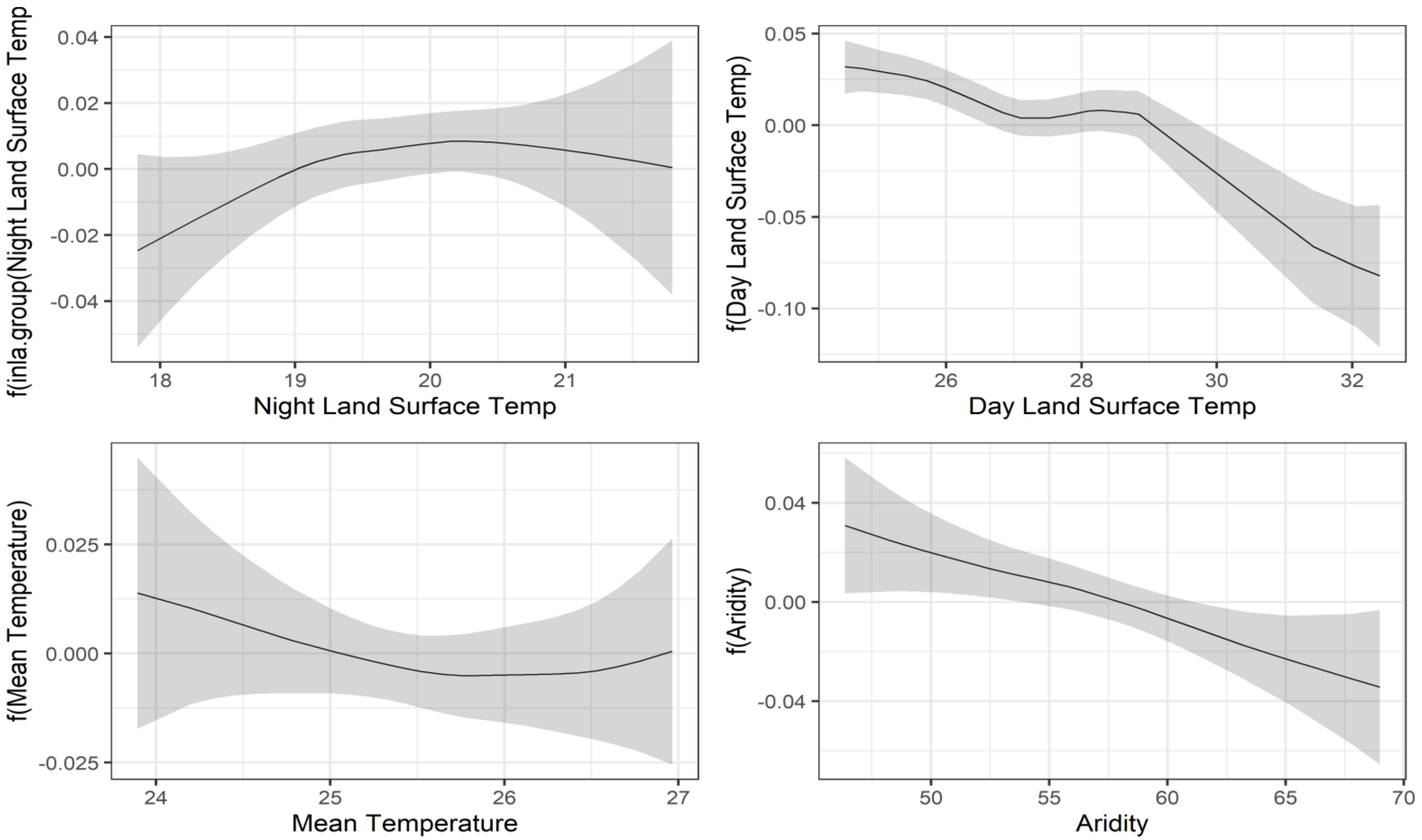


***Figure 19**: GAM on summarized data without year effect*

***Table 17**: Checking spatial autocorrelation on the residuals*

| Model | Statistic | P-value | Method | Alternative |
|---|---|---|---|---|
| SEM | -0.07 | 0.81 | Monte-Carlo simulation of Moran I | greater |
| SLM | 0.15 | 0.03 | Monte-Carlo simulation of Moran I | greater |
| SDM | -0.03 | 0.68 | Monte-Carlo simulation of Moran I | greater |
| SDEM | -0.03 | 0.64 | Monte-Carlo simulation of Moran I | greater |

***Table 18***: *DIC for all GAMs*

| Model | Linear relation variables with rho estimated | SGAM nonlinear all environment variables with rho = 0 | SGAM nonlinear all environment variables with rho estimated | SGAM mixed linear nonlinear with rho estimated |
|---|---|---|---|---|
| M1 | -4435.66 | -3668.78 | -6878.09 | -6878.10 |
| M2 | -4565.98 | -3680.14 | -7746.42 | -7544.43 |
| M3 | -4325.32 | -4320.39 | -6748.46 | -6811.30 |
| M4 | -4497.76 | -4385.93 | -7888.71 | -7801.80 |

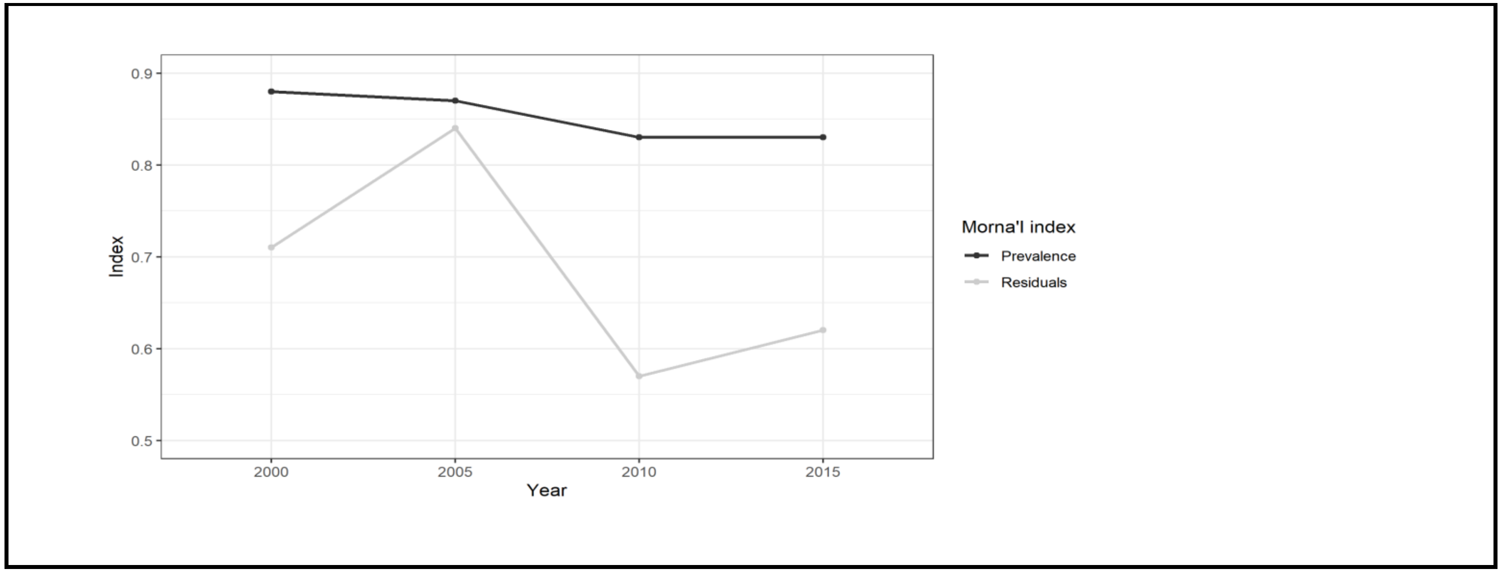


**Figure 20**: Spatial autocorrelation on residuals and malaria prevalence over time. There was overwhelming evidence of the observed spatial autocoorelation (P < 0.001).

***Table 19**: Comparison of rho from ML vs INLA*

| Method | Mean rho ML | P-value[a] | P-value[b] | AIC ML | Mean rho INLA (SD) | 95% CI | DIC |
|---|---|---|---|---|---|---|---|
| SEM | 0.51 | <0.001 | <0.001 | -1242 | 0.50 (0.04) | 0.42-0.58 | -3125.57 |
| SLM | 0.35 | <0.001 | <0.001 | -1210 | 0.84 (0.00) | 0.84-0.85 | -3125.46 |
| SDM | 0.50 | <0.001 | <0.001 | -1250 | 0.47 (0.03) | 0.39-0.52 | -3125.57 |
| SDEM | 0.51 | <0.001 | <0.001 | -1252 | 0.50 (0.04) | 0.42-0.58 | -3125.58 |

[a]P-value from Likelihood Ratio test;

[b]P-value from Wald test. Both tests are for evaluating if the spatial correlation coefficient is different from 0
